# Novel Small Molecules Capable of Blocking mtRAS-Signaling Pathway

**DOI:** 10.3389/fonc.2021.768022

**Published:** 2021-12-09

**Authors:** Namkyoung Kim, Injae Shin, Younghoon Kim, Eunhye Jeon, Jiwon Lee, Chaeyoung Lee, Yunju Nam, Sumin Lee, Eunhye Ju, Chan Kim, Woolim Son, SeongShick Ryu, Minjoo Ko, Taebo Sim

**Affiliations:** ^1^ KU-KIST Graduate School of Converging Science and Technology, Korea University, Seoul, South Korea; ^2^ Severance Biomedical Science Institute, Graduate School of Medical Science, Yonsei University College of Medicine, Seoul, South Korea

**Keywords:** RAS signaling pathway, cancers with mtRAS, mutated RAS, RAS signaling blocker, multi-targeted kinase inhibitor, GNF-7

## Abstract

RAS mutants are involved in approximately 30% of all human cancers and have been regarded as undruggable targets owing to relatively smooth protein surface and obscure binding pockets. In our previous study, we have demonstrated that GNF-7, a multi-targeted kinase inhibitor, possesses potent anti-proliferative activity against Ba/F3 cells transformed with NRAS-G12D. Based on our further analysis using Ba/F3 cells transformed with mtRAS, we discovered a series of pyrimido[4,5-*d*]pyrimidin-2-one analogues as mtRAS-signaling pathway blockers. In addition, our efforts expanded the assessment to cancer cells with mtRAS, which revealed that these substances are also capable of strongly suppressing the proliferation of various cancer cells harboring KRAS-G12D (AsPC-1), KRAS-G12V (SW480, DU-145), KRAS-G12C (H358), KRAS-G13D (MDA-MB-231), KRAS-Q61L (HT-29), and NRAS-Q61L (OCI-AML3). We herein report novel and potent mtRAS-signaling pathway blockers, SIJ1795 and SIJ1772, possessing 2 to 10-fold increased anti-proliferative activities compared to those of GNF-7 on cancer cells harboring mtRAS as well as on Ba/F3 cells transformed with mtRAS. Both SIJ1795 and SIJ1772 attenuate phosphorylation of RAS downstream molecules (AKT and MEK) and induce apoptosis and G0/G1 cell cycle arrest on cancer cells with mtRAS. Moreover, both substances substantially suppress the migration, invasion, and colony formation of cancer cells harboring mtRAS. Taken together, this study led us to identification of SIJ1795 and SIJ1772 capable of strongly inhibiting mtRAS-signaling pathway on cancer cells harboring mtRAS.

## 1 Introduction

RAS proteins are small GTPase, which function as a binary molecular switch controlling the signal transduction by switching between active and inactive states. The equilibrium is shifted to guanosine triphosphate (GTP)-bound RAS active state when GDP/GTP exchange occurs by guanine nucleotide exchange factor (GEF). On the other hand, guanosine diphosphate (GDP)-bound RAS inactive state is formed through GTPase-activating protein (GAP)-mediated hydrolysis of GTP (1). As is well known, RAS proteins are notorious for possessing extremely high affinity toward GTP and relatively obscure binding pockets contributing to poor interaction with small molecules (2). Moreover, mutations of RAS proteins at codons 12, 13, and 61 interfere with GAP-mediated GTP hydrolysis consequently resulting in constitutively activated GTP-bound RAS forms (3, 4). Thus formed RAS active state stimulates the downstream signaling pathways such as MAPK (RAS-RAF-MEK-ERK) (5) and PI3K (PI3K-AKT-mTOR) pathways closely associated with cell cycle, growth, proliferation, survival, and differentiation (3–6).

Mutated RAS (mtRAS) is often regarded as the most common oncogene and found in approximately 30% of human tumors comprising various cancers such as pancreatic cancer (90%), colon cancer (45%), and lung cancer (35%) (7). RAS is composed of three isoforms: KRAS, NRAS, and HRAS. KRAS is the most commonly mutated (86%), followed by NRAS (11%), and HRAS (3%) in RAS-driven human cancers (8, 9). The most common mutants of KRAS are G12 mutation (83%), G13 mutation (14%), and Q61 mutation (2%), of NRAS are Q61 mutation (62%), G12 mutation (23%), and of HRAS are G12 mutation (35%), Q61 mutation (34%), G13 mutation (27%) (8–10). AMG-510 (sotorasib) is a first-in-class drug approved by FDA in 2021 for treatment of non-small cell lung cancer (NSCLC) harboring KRAS-G12C mutant in adults. However, sotorasib can only target KRAS-G12C mutation by forming covalent bond with Cys12 (11). Although many recent studies are focused on targeting mtRAS through protein–protein interaction (PPI) (12–16), protein degradation (17–19), combination strategies (3, 20–24), and single-agent inhibitor (3), there are still no approved drugs for mtRAS other than AMG-510 (sotorasib) targeting only KRAS-G12C.

Previously, we reported that dual inhibition of activated cdc42-associated kinase 1 (ACK1) and germinal center kinase (GCK) can be a novel therapeutic strategy to overcome acute myeloid leukemia (AML) harboring NRAS mutation (25). Multi-targeted kinase inhibitor GNF-7 (26) and its derivatives are shown to be able to suppress proliferation of both Ba/F3 cells transformed with NRAS-G12D and human AML cell lines, OCI-AML3 (NRAS-Q61L) *via* dual inhibition of ACK1 and GCK. Moreover, GNF-7 and its derivatives displayed effect on anti-leukemic efficacies in blood circulating model (Ba/F3-NRAS-G12D) and xenograft model (OCI-AML3) as well as AKT/mTOR and GCK signaling, apoptosis, cell cycle arrest, anchorage independent growth in Ba/F3-NRAS-G12D and OCI-AML3 (27). GNF-7 and its derivatives are also reported as pan-class (class I/II/III) BRAF inhibitor in our previous studies, indicating that inhibiting BRAF may be helpful for inhibiting mtRAS signaling pathway (28, 29). Overall, our previous findings strongly suggest that the use of multi-targeted kinase inhibitor such as GNF-7 can provide an effective approach to discover novel inhibitors targeting mtRAS *via* blocking its downstream signaling pathway.

In the current effort, we discovered that GNF-7 and its derivatives possess potent anti-proliferative activities against Ba/F3 cells transformed with mtRAS and suppress MAPK and AKT downstream signals. It is noteworthy that, based on our results, GNF-7 and its derivatives are capable of blocking strongly mtRAS signal pathway. Encouraged by these findings, we have expanded the assessment to various cancer cells harboring KRAS G12D (AsPC-1), KRAS-G12V (SW480, DU-145), KRAS-G12C (H358), KRAS-G13D (MDA-MB-231), KRAS-Q61L (HT-29), and NRAS-Q61L (OCI-AML3). Especially, SIJ1795 and SIJ1772 exhibited 2- to 10-fold much higher anti-proliferative activities on all cancer cells harboring mtRAS compared with GNF-7 (GI_50_s = 0.029 to 0.447 µM). Moreover, SIJ1795 and SIJ1772 significantly suppressed proliferation, migration, invasion, and colony formation of cancer cells harboring mtRAS.

## 2 Materials and Methods

### 2.1 Chemistry

#### 2.1.1 General Information

Unless otherwise described, all commercial reagents and solvents were purchased from commercial suppliers and used without further purification. All reactions were performed under a N_2_ atmosphere in flame-dried glassware. Reactions were monitored by using TLC with 0.25 mm E. Merck precoated silica gel plates (60 F254). Reaction progress was monitored by using TLC analysis using a UV lamp, ninhydrin, or *p*-anisaldehyde stain for detection purposes. All solvents were purified by using standard techniques. Purification of reaction products was carried out by using silica gel column chromatography with Kieselgel 60 Art. 9385 (230–400 mesh). Purities of all compounds were >95%, and mass spectra and purities of all compounds was accessed using Waters LC/MS system (Waters QDA Detector, Waters 2998 Photodiode Array Detector, Waters SFO System Fluidics Organizer, Water 2545 Binary Gradient Module, Waters 2767 Sample Manager) using SunFire™ C_18_ column (4.6 × 50 mm, 5 μm particle size): solvent gradient = 90% A at 0 min, 0% A at 5 min. Solvent A = 0.10% TFA in H_2_O; Solvent B = 0.10% TFA in MeOH; flow rate: 0.8 ml/min. ^1^H and ^13^C NMR spectra were obtained using Bruker 400 MHz FT-NMR (400 MHz for ^1^H, and 100 MHz for ^13^C) spectrometer and Bruker 300 MHz FT-NMR (300 MHz for ^1^H, and 75.5 MHz for ^13^C). Standard abbreviations are used for denoting the signal multiplicities.

*N*-(4-Methyl-3-(1-methyl-2-oxo-7-((4-(piperidin-1-yl)phenyl)amino)-1,4-dihydropyrimido[4,5-*d*]pyrimidin-3(2*H*)-yl)phenyl)-3-(trifluoromethyl)benzamide (1). To a solution of *N*-(3-(7-chloro-1-methyl-2-oxo-1,4-dihydropyrimido[4,5-*d*]pyrimidin-3(*2H*)-yl)-4-methylphenyl)-3-(trifluoromethyl)benzamide (27) (100 mg, 0.211 mmol) in 2-butanol (2 ml) was added 4-(piperidin-1-yl)aniline (41 mg, 0.232 mmol), K_2_CO_3_ (690 mg, 1.055 mmol), Xphos (20 mg, 0.042 mmol) and Pd_2_(dba)_3_ (40 mg, 0.042 mmol) at room temperature. The reaction mixture was then stirred for 1 h at 100°C, cooled to room temperature, filtered and concentrated. The resulting residue was purified by silica gel column chromatography on silica gel (10 to 50% THF/hexane) to afford 1 (92 mg, 71%) as a brown solid. ^1^H NMR (300 MHz, DMSO-*d*_6_) δ 10.52 (s, 1H), 9.29 (s, 1H), 8.31–8.25 (m, 2H), 8.09 (s, 1H), 7.97 (d, *J* = 8.0 Hz, 1H), 7.82–7.76 (m, 2H), 7.65 (dd, *J* = 1.9 Hz, 8.3 Hz, 1H), 7.59–7.56 (m, 2H), 7.31 (d, *J* = 8.3 Hz, 1H), 6.89–6.86 (d, *J* = 9.1 Hz, 2H), 4.68 (d, *J* = 14.0 Hz, 1H), 4.49 (d, *J* = 14.0 Hz, 1H), 3.5–3.02 (m, 4H), 2.14 (s, 3H), 1.66–1.61 (m, 4H), 1.52–1.50 (m, 2H); ^13^C NMR (75.5 MHz, DMSO-*d*_6_) δ 163.9, 159.2, 156.9, 153.2, 152.3, 147.0, 141.3, 137.6, 135.6, 132.5, 131.8, 131.0, 130.8, 129.8, 129.4, 129.0, 128.2, 125.8, 124.2, 124.2, 122.2, 120.2, 119.8, 119.4, 116.5, 101.8, 50.5, 46.7, 28.2, 25.5, 23.9, 16.8. LCMS (ESI) *m/z* 616 [M + H]^+^.

*N*-(3-(7-((4-(4-Hydroxypiperidin-1-yl)phenyl)amino)-1-methyl-2-oxo-1,4-dihydropyrimido[4,5-*d*]pyrimidin-3(2*H*)-yl)-4-methylphenyl)-3-(trifluoromethyl)benzamide (2). To a solution of *N*-(3-(7-chloro-1-methyl-2-oxo-1,4-dihydropyrimido[4,5-*d*]pyrimidin-3(2*H*)-yl)-4-methylphenyl)-3-(trifluoromethyl)benzamide (27) (100 mg, 0.211 mmol) in 2-butanol (2 ml) was added 1-(4-aminophenyl)piperidin-4-ol (45 mg, 0.232 mmol), K_2_CO_3_ (690 mg, 1.055 mmol), Xphos (20 mg, 0.042 mmol), and Pd_2_(dba)_3_ (40 mg, 0.042 mmol) at room temperature. The reaction mixture was then stirred for 1 h at 100°C, cooled to room temperature, filtered and concentrated. The resulting residue was purified by silica gel column chromatography on silica gel (0 to 5% MeOH/DCM) to afford 2 (85 mg, 64%) as a pale brown solid. ^1^H NMR (400 MHz, DMSO-*d*_6_) δ 10.55 (s, 1H), 9.33 (s, 1H), 8.30 (s, 1H), 8.26 (d, *J* = 7.8 Hz, 1H), 8.09 (s, 1H), 7.98 (d, *J* = 8.1 Hz, 1H), 7.81–7.78 (m, 2H), 7.64 (dd, *J* = 2.0, 8.3 Hz, 1H), 7.58–7.55 (m, *J* = 9.1 Hz, 2H), 7.31 (d, *J* = 8.3 Hz, 1H), 6.90–6.87 (m, 2H), 4.69–4.66 (m, 2H), 4.49 (d, *J* = 13.9 Hz, 1H), 3.62–3.54 (m, 1H), 3.44–3.41 (m, 2H), 3.32 (s, 3H), 2.77–2.71 (m, 2H), 2.13 (s, 3H), 1.83–1.80 (m, 2H), 1.52–1.43 (m, 2H); ^13^C NMR (100 MHz, DMSO-d_6_) δ 163.9, 159.2, 156.9, 153.2, 152.3, 146.2, 141.3, 137.6, 135.6, 132.3, 131.8, 131.0, 130.8, 129.8, 129.7, 129.4, 129.0, 128.7, 128.2, 128.2, 125.4, 124.2, 124.2, 122.6, 120.2, 119.8, 119.3, 116.3, 66.1, 47.4, 46.7, 34.0, 28.2, 16.8. LCMS (ESI) *m/z* 632 [M + H]^+^.

*N*-(4-Methyl-3-(1-methyl-7-((4-morpholinophenyl)amino)-2-oxo-1,4-dihydropyrimido[4,5-*d*]pyrimidin-3(2*H*)-yl)phenyl)-3-(trifluoromethyl)benzamide (3). To a solution of *N*-(3-(7-chloro-1-methyl-2-oxo-1,4-dihydropyrimido[4,5-*d*]pyrimidin-3(2*H*)-yl)-4-methylphenyl)-3-(trifluoromethyl)benzamide (27) (100 mg, 0.211 mmol) in 2-butanol (2 ml) was added 4-morpholinoaniline (41 mg, 0.232 mmol), K_2_CO_3_ (690 mg, 1.055 mmol), Xphos (20 mg, 0.042 mmol) and Pd_2_(dba)_3_ (40 mg, 0.042 mmol) at room temperature. The reaction mixture was then stirred for 1 h at 100°C, cooled to room temperature, filtered and concentrated. The resulting residue was purified by silica gel column chromatography on silica gel (10 to 50% THF/hexane) to afford **3** (71 mg, 55%) as a gray solid. ^1^H NMR (400 MHz, DMSO-*d*_6_) δ 10.55 (s, 1H), 9.37 (s, 1H), 8.31 (s, 1H), 8.26 (d, *J* = 7.8 Hz, 1H), 8.10 (s, 1H), 7.98 (d, *J* = 8.1 Hz, 1H), 7.81–7.78 (m, 2H), 7.65–7.60 (m, 3H), 7.32 (d, *J* = 8.6 Hz, 1H), 6.90 (d, *J* = 9.1 Hz, 2H), 4.68 (d, *J* = 13.9 Hz, 1H), 4.50 (d, *J* = 14.2 Hz, 1H), 3.74–3.72 (m, 4H), 3.33 (s, 3H), 3.04–3.02 (m, 4H), 2.14 (s, 3H); ^13^C NMR (75.5 MHz, DMSO-*d*_6_) δ 163.8, 159.2, 156.9, 153.2, 152.2, 146.1, 141.2, 137.6, 135.6, 133.0, 131.8, 130.9, 130.7, 129.8, 129.4, 129.0, 128.2, 128.2, 128.1, 128.1, 125.8, 124.2, 124.1, 122.2, 120.1, 119.8, 119.3, 115.6, 101.8, 66.2, 49.3, 46.7, 28.2, 16.8. LCMS (ESI) *m/z* 618 [M + H]^+^.

*N*-(3-(7-((4-(4-Ethylpiperazin-1-yl)phenyl)amino)-1-methyl-2-oxo-1,4-dihydropyrimido[4,5-*d*]pyrimidin-3(2*H*)-yl)-4-methylphenyl)-3-(trifluoromethyl)benzamide (4). The synthesis of 4 is described in our previous report (27).

*N*-(4-Methyl-3-(1-methyl-7-((3-morpholinopropyl)amino)-2-oxo-1,4-dihydropyrimido[4,5-*d*]pyrimidin-3(2*H*)-yl)phenyl)-3-(trifluoromethyl)benzamide (5). To a solution of *N*-(3-(7-chloro-1-methyl-2-oxo-1,4-dihydropyrimido[4,5-*d*]pyrimidin-3(2*H*)-yl)-4-methylphenyl)-3-(trifluoromethyl)benzamide (27) (100 mg, 0.211 mmol) in DMF (2 ml) was added 3-morpholinopropan-1-amine (60 mg, 0.422 mmol), K_2_CO_3_ (87 mg, 0.633 mmol) at room temperature. The reaction mixture was then stirred for 5 h at 100°C, cooled to room temperature, diluted with ethyl acetate and washed with brine, dried over MgSO4, filtered and concentrated. The resulting residue was purified by silica gel column chromatography on silica gel (0 to 5% MeOH/DCM) to afford **5** (100 mg, 82%) as a white solid. ^1^H NMR (400 MHz, DMSO-*d*_6_) δ 10.53 (s, 1H), 8.30 (s, 1H), 8.26 (d, *J* = 7.8 Hz, 1H), 7.99–7.96 (m, 2H), 7.79 (t, *J* = 7.8 Hz, 1H), 7.76 (d, *J* = 2.0 Hz, 1H), 7.63 (dd, *J* = 2.1, 8.44 Hz, 1H), 7.30 (d, *J* = 8.6 Hz, 1H), 7.17 (m, 1H), 4.60 (d, *J* = 13.7 Hz, 1H), 4.41 (d, *J* = 13.9 Hz, 1H), 3.57 (t, *J* = 4.4 Hz, 4H), 3.30 (m, 2H), 3.26 (s, 3H), 2.35–2.31 (m, 6H), 2.11 (s, 3H), 1.69 (m, 2H); ^13^C NMR (75.5 MHz, DMSO-*d*_6_) δ 163.8, 161.6, 156.8, 152.4, 141.3, 137.6, 135.6, 131.8, 130.9, 130.7, 129.8, 129.7, 129.4, 129.0, 128.5, 128.2, 128.2, 128.1, 128.1, 125.8, 124.2, 124.2, 124.1, 124.1, 122.2, 119.7, 119.3, 66.2, 56.2, 53.4, 46.6, 27.7, 25.9, 16.8. LCMS (ESI) *m/z* 584 [M + H]^+^.

*N*-(3-(7-((2,6-Dimethylpyridin-3-yl)amino)-1-methyl-2-oxo-1,4-dihydropyrimido[4,5-*d*]pyrimidin-3(2*H*)-yl)-4-methylphenyl)-3-(trifluoromethyl)benzamide (6). To a solution of *N*-(3-(7-chloro-1-methyl-2-oxo-1,4-dihydropyrimido[4,5-*d*]pyrimidin-3(2*H*)-yl)-4-methylphenyl)-3-(trifluoromethyl)benzamide (27) (100 mg, 0.211 mmol) in 2-butanol (2 ml) was added 2,6-dimethylpyridin-3-amine (28 mg, 0.232 mmol), K_2_CO_3_ (690 mg, 1.055 mmol), Xphos (20 mg, 0.042 mmol) and Pd_2_(dba)_3_ (40 mg, 0.042 mmol) at room temperature. The reaction mixture was then stirred for 1 h at 100°C, cooled to room temperature, filtered and concentrated. The resulting residue was purified by silica gel column chromatography on silica gel (30 to 70% THF/hexane) to afford **6** (86 mg, 73%) as a pale gray solid. ^1^H NMR (400 MHz, DMSO-*d*_6_) δ 10.55 (s, 1H), 8.34 (s, 1H), 8.30 (s, 1H), 8.26 (d, *J* = 8.0 Hz, 1H), 7.98 (d, *J* = 7.8 Hz, 1H), 7.83 (d, *J* = 2.1 Hz, 1H), 7.82–7.78 (m, 1H), 7.64 (dd, *J* = 2.2, 8.2 Hz, 1H), 7.33 (d, *J* = 8.6 Hz, 1H), 4.83 (dd, *J* = 1.0, 14.9 Hz, 1H), 4.67 (d, *J* = 15.7 Hz, 1H), 3.29 (s, 3H), 2.14 (s, 3H). ^13^C NMR (100 MHz, DMSO-*d*_6_) δ 163.8, 159.9, 157.0, 153.2, 152.4, 152.1, 151.6, 141.2, 137.5, 135.6, 132.6, 131.7, 131.3, 130.9, 130.6, 129.7, 129.3, 129.0, 128.1, 125.3, 124.2, 124.1, 124.1, 124.0, 122.6, 120.2, 119.7, 119.3, 102.3, 46.6, 27.8, 23.4, 21.0, 16.7. LCMS (ESI) *m/z* 562 [M + H]^+^.

*N*-(3-(7-((2-Methoxy-4-morpholinophenyl)amino)-1-methyl-2-oxo-1,4-dihydropyrimido[4,5-*d*]pyrimidin-3(2*H*)-yl)-4-methylphenyl)-3-(trifluoromethyl)benzamide (**7**). To a solution of *N*-(3-(7-chloro-1-methyl-2-oxo-1,4-dihydropyrimido[4,5-*d*]pyrimidin-3(2*H*)-yl)-4-methylphenyl)-3-(trifluoromethyl)benzamide (27) (100 mg, 0.211 mmol) in 2-butanol (2 ml) was added 2-methoxy-4-morpholinoaniline (48 mg, 0.232 mmol), K_2_CO_3_ (690 mg, 1.055 mmol), Xphos (20 mg, 0.042 mmol) and Pd_2_(dba)_3_ (40 mg, 0.042 mmol) at room temperature. The reaction mixture was then stirred for 1 h at 100°C, cooled to room temperature, filtered and concentrated. The resulting residue was purified by silica gel column chromatography on silica gel (30 to 70% THF/hexane) to afford **7** (87 mg, 71%) as a pale brown solid. ^1^H NMR (400 MHz, DMSO-*d*_6_) δ 10.52 (s, 1H), 8.30 (s, 1H), 8.26 (d, *J* = 7.8 Hz, 1H), 8.05 (s, 1H), 7.97 (d, *J* = 7.8 Hz, 1H), 7.89 (s, 1H), 7.82–7.77 (m, 3H), 7.64 (dd, *J* = 2.1, 8.3 Hz, 1H), 7.31 (d, *J* = 8.6 Hz, 1H), 6.65 (d, *J* = 2.5 Hz, 1H), 6.50 (dd, *J* = 2.5, 8.8 Hz, 1H), 4.67 (d, *J* = 14.2 Hz, 1H), 4.49 (d, *J* = 14.2 Hz, 1H), 3.83 (s, 3H), 3.75–3.73 (m, 4H), 3.28 (s, 3H), 3.10–3.08 (m, 4H), 2.13 (s, 3H); ^13^C NMR (75.5 MHz, DMSO-*d*_6_) δ 163.9, 159.4, 156.9, 153.2, 152.2, 150.7, 147.9, 141.2, 137.6, 135.6, 131.8, 130.9, 130.7, 129.8, 129.8, 129.4, 129.0, 128.6, 128.2, 128.1, 125.8, 124.2, 124.2, 124.1, 124.1, 122.2, 122.1, 120.9, 119.8, 119.3, 106.6, 102.1, 99.9, 66.2, 55.7, 49.2, 46.6, 28.0, 16.8. LCMS (ESI) *m/z* 648 [M + H]^+^.

*N*-(3-(7-((4-(4-Ethylpiperazin-1-yl)-2-(trifluoromethyl)phenyl)amino)-1-methyl-2-oxo-1,4-dihydropyrimido[4,5-*d*]pyrimidin-3(2*H*)-yl)-4-methylphenyl)-3-(trifluoromethyl)benzamide (8). To a solution of *N*-(3-(7-chloro-1-methyl-2-oxo-1,4-dihydropyrimido[4,5-*d*]pyrimidin-3(2*H*)-yl)-4-methylphenyl)-3-(trifluoromethyl)benzamide (27) (100 mg, 0.211 mmol) in 2-butanol (2 ml) was added 4-(4-ethylpiperazin-1-yl)-2-(trifluoromethyl)aniline (63 mg, 0.232 mmol), K_2_CO_3_ (690 mg, 1.055 mmol), Xphos (20 mg, 0.042 mmol) and Pd_2_(dba)_3_ (40 mg, 0.042 mmol) at room temperature. The reaction mixture was then stirred for 1 h at 100°C, cooled to room temperature, filtered and concentrated. The resulting residue was purified by silica gel column chromatography on silica gel (20 to 60% THF/hexane) to afford **8** (84 mg, 56%) as a pale brown solid. ^1^H NMR (400 MHz, DMSO-*d*_6_) δ 10.53 (s, 1H), 8.57 (s, 1H), 8.30 (s, 1H), 8.26 (d, *J* = 7.8 Hz, 1H), 7.98–7.97 (m, 2H), 7.81–7.76 (m, 2H), 7.63 (dd, *J* = 1.9, 8.4 Hz, 1H), 7.38 (d, *J* = 8.8 Hz, 1H), 7.30 (d, *J* = 8.5 Hz, 1H), 7.22 (dd, *J* = 2.3, 9.03 Hz, 1H), 7.13 (d, *J* = 2.3 Hz, 1H), 4.65 (d, *J* = 14.1 Hz, 1H), 4.46 (d, *J* = 14.1 Hz, 1H), 3.20 (s, 7H), 2.37 (q, *J* = 7.2 Hz, 2H), 2.12 (s, 3H), 1.04 (t, *J* = 7.2 Hz, 3H). ^13^C NMR (100 MHz, DMSO-*d*_6_) δ 163.9, 161.2, 157.0, 153.2, 152.2, 148.7, 141.3, 137.6, 135.6, 131.8, 131.3, 130.9, 130.7, 129.8, 129.7, 129.4, 129.0, 128.7, 128.2, 128.2, 127.8, 126.0, 125.8, 125.3, 124.2, 124.2, 124.2, 124.1, 122.6, 119.8, 119.3, 119.1, 111.7, 111.7, 111.6, 111.6, 102.0, 52.2, 51.6, 47.9, 46.6, 27.8, 16.8, 12.0. LCMS (ESI) *m/z* 713 [M + H]^+^.

*N*-(3-(7-((3-(4-Ethylpiperazin-1-yl)phenyl)amino)-1-methyl-2-oxo-1,4-dihydropyrimido[4,5-*d*]pyrimidin-3(2*H*)-yl)-4-methylphenyl)-3-(trifluoromethyl)benzamide (SIJ1795). To a solution of *N*-(3-(7-chloro-1-methyl-2-oxo-1,4-dihydropyrimido[4,5-*d*]pyrimidin-3(2*H*)-yl)-4-methylphenyl)-3-(trifluoromethyl)benzamide (27) (100 mg, 0.211 mmol) in 2-butanol (2 ml) was added 3-(4-ethylpiperazin-1-yl)aniline (48 mg, 0.232 mmol), K_2_CO_3_ (690 mg, 1.055 mmol), Xphos (20 mg, 0.042 mmol) and Pd_2_(dba)_3_ (40 mg, 0.042 mmol) at room temperature. The reaction mixture was then stirred for 1 h at 100°C, cooled to room temperature, filtered and concentrated. The resulting residue was purified by silica gel column chromatography on silica gel (0 to 10% MeOH/DCM) to afford SIJ1795 (105 mg, 77%) as a white solid. ^1^H NMR (400 MHz, acetone-*d*_6_) δ 9.92 (s, 1H), 8.53 (s, 1H), 8.32–8.29 (m, 2H), 8.12 (s, 1H), 7.92 (d, *J* = 7.8 Hz, 1H), 7.88 (d, *J* = 2.2 Hz, 1H), 7.79–7.75 (m, 1H), 7.67 (dd, *J* = 2.2, 8.3 Hz, 1H), 7.59 (t, *J* = 2.1 Hz, 1H), 7.29–7.24 (m, 2H), 7.17–7.13 (m, 1H), 6.60 (dd, *J* = 1.8, 8.2 Hz, 1H), 4.78 (d, *J* = 14.4 Hz, 1H), 4.57 (d, *J* = 14.2 Hz, 1H), 3.41 (s, 3H), 3.22–3.20 (m, 4H), 2.57–2.54 (m, 4H), 2.40 (q, *J* = 7.2 Hz, 2H), 2.18 (s, 3H), 1.07 (t, *J* = 7.2 Hz, 3H); ^13^C NMR (100 MHz, DMSO-*d*_6_) δ 163.8, 159.1, 156.8, 153.2, 152.2, 151.4, 141.2, 141.2, 137.6, 135.6, 131.8, 130.9, 130.7, 129.7, 129.4, 129.1, 128.8, 128.2, 128.1, 128.0, 125.3, 124.2, 124.2, 124.2, 124.1, 122.6, 119.9, 119.8, 119.4, 109.9, 108.9, 106.0, 102.3, 52.4, 51.6, 48.4, 46.7, 28.2, 16.8, 11.9. LCMS (ESI) *m/z* 645 [M + H]^+^.

*N*-(4-Methyl-3-(1-methyl-7-((1-methyl-1*H*-pyrazol-4-yl)amino)-2-oxo-1,4-dihydropyrimido[4,5-*d*]pyrimidin-3(2*H*)-yl)phenyl)-3-(trifluoromethyl)benzamide (10). The synthesis of 10 is described in our previous report (27).

*N*-(4-Methyl-3-(1-methyl-7-((1-methyl-1*H*-pyrazol-3-yl)amino)-2-oxo-1,4-dihydropyrimido[4,5-*d*]pyrimidin-3(2*H*)-yl)phenyl)-3-(trifluoromethyl)benzamid (11). The synthesis of 11 is described in our previous report (29).

*N*-(3-(7-((1-(2-Methoxyethyl)-1*H*-pyrazol-4-yl)amino)-1-methyl-2-oxo-1,4-dihydropyrimido[4,5-*d*]pyrimidin-3(2*H*)-yl)-4-methylphenyl)-3-(trifluoromethyl)benzamide (12). To a solution of *N*-(3-(7-chloro-1-methyl-2-oxo-1,4-dihydropyrimido[4,5-*d*]pyrimidin-3(2*H*)-yl)-4-methylphenyl)-3-(trifluoromethyl)benzamide (27) (100 mg, 0.211 mmol) in 2-butanol (2 ml) was added 1-(2-methoxyethyl)-1*H*-pyrazol-4-amine (33 mg, 0.232 mmol), K_2_CO_3_ (690 mg, 1.055 mmol), Xphos (20 mg, 0.042 mmol) and Pd_2_(dba)_3_ (40 mg, 0.042 mmol) at room temperature. The reaction mixture was then stirred for 1 h at 100°C, cooled to room temperature, filtered and concentrated. The resulting residue was purified by silica gel column chromatography on silica gel (30 to 70% THF/hexane) to afford 12 (94 mg, 77%) as a pale brown solid. ^1^H NMR (400 MHz, DMSO-*d*_6_) δ 10.53 (s, 1H), 9.43 (bs, 1H), 8.30 (s, 1H), 8.26 (d, *J* = 8.1 Hz, 1H), 8.10 (s, 1H), 7.97 (d, *J* = 7.8 Hz, 1H), 7.91 (s, 1H), 7.81–7.78 (m, 2H), 7.64 (dd, *J* = 2.0, 8.3 Hz, 1H), 7.52 (s, 1H), 7.31 (d, *J* = 8.6 Hz, 1H), 4.68 (d, *J* = 13.9 Hz, 1H), 4.49 (d, *J* = 13.9 Hz, 1H), 4.22 (t, *J* = 5.1 Hz, 2H), 3.66 (t, *J* = 5.3 Hz, 2H), 3.24 (s, 3H), 2.13 (s, 3H); ^13^C NMR (100 MHz, DMSO-*d*_6_) δ 163.9, 158.6, 157.1, 152.3, 141.3, 137.6, 135.6, 131.8, 130.9, 130.8, 129.9, 129.8, 129.7, 129.4, 129.0, 128.7, 128.2, 128.2, 125.4, 124.2, 124.2, 124.2, 124.1, 123.1, 122.6, 120.0, 120.0, 119.9, 119.9, 119.8, 119.3, 70.8, 58.0, 51.3, 46.7, 28.3, 16.8. LCMS (ESI) *m/z* 581 [M + H]^+^.

*N*-(3-(7-((1-(1-Ethylpiperidin-4-yl)-1*H*-pyrazol-4-yl)amino)-1-methyl-2-oxo-1,4-dihydropyrimido[4,5-*d*]pyrimidin-3(2*H*)-yl)-4-methylphenyl)-3-(trifluoromethyl)benzamide (13). The synthesis of 13 is described in our previous report (28).

*N*-(4-Methyl-3-(1-methyl-7-((1-(1-(methylsulfonyl)piperidin-4-yl)-1*H*-pyrazol-4-yl)amino)-2-oxo-1,4-dihydropyrimido[4,5-*d*]pyrimidin-3(2*H*)-yl)phenyl)-3-(trifluoromethyl)benzamide (SIJ1772). To a solution of *N*-(3-(7-chloro-1-methyl-2-oxo-1,4-dihydropyrimido[4,5-*d*]pyrimidin-3(2*H*)-yl)-4-methylphenyl)-3-(trifluoromethyl)benzamide (27) (100 mg, 0.211 mmol) in 2-butanol (2 ml) was added 1-(1-(methylsulfonyl)piperidin-4-yl)-1*H*-pyrazol-4-amine (57 mg, 0.232 mmol), K_2_CO_3_ (690 mg, 1.055 mmol), Xphos (20 mg, 0.042 mmol) and Pd_2_(dba)_3_ (40 mg, 0.042 mmol) at room temperature. The reaction mixture was then stirred for 1 h at 100°C, cooled to room temperature, filtered and concentrated. The resulting residue was purified by silica gel column chromatography on silica gel (0 to 5% MeOH/DCM) to afford SIJ1772 (30 mg, 21%) as a pale gray solid. ^1^H NMR (300 MHz, DMSO-*d*_6_) δ 10.54 (s, 1H), 9.45 (bs, 1H), 8.36–8.25 (m, 2H), 8.11 (s, 1H), 8.03–7.92 (m, 2H), 7.84–7.77 (m, 2H), 7.66 (d, *J* = 8.4 Hz, 1H), 7.59 (s, 1H), 7.32 (d, *J* = 8.4 Hz, 1H), 4.69 (d, *J* = 14.2 Hz, 1H), 4.51 (d, *J* = 13.9 Hz, 1H), 4.37-4.25 (m, 1H), 3.67 (d, *J* = 11.5 Hz, 2H), 3.37 (s, 3H), 2.98–2.91 (m, 2H), 2.93 (s, 3H), 2.15 (s, 3H), 2.11 (m, 2H), 2.03–1.89 (m, 2H); ^13^C NMR (100 MHz, DMSO-*d*_6_) δ 163.9, 158.7, 157.2, 152.3, 141.3, 137.6, 135.6, 131.9, 130.9, 130.8, 129.8, 129.4, 129.0, 128.7, 128.2, 125.4, 124.2, 123.1, 122.7, 119.8, 119.3, 57.2, 46.7, 44.6, 34.6, 31.5, 16.9. LRMS (ESI) *m/z* 684 [M+H]^+^.

### 2.2 Biology

#### 2.2.1 Cell Culture

Ba/F3-parental, OCI-AML3, U397 cells were purchased from DSMZ. MDA-MB-231, HCT-116, AsPC-1, H358, HT-29, SW480, and DU-145 cells were obtained from KCLB. MDA-MB-468 cells were obtained from ATCC. All cells were maintained in RPMI with 10% FBS and antibiotics. Cells were grown in a humidified 5% CO_2_ incubator at 37°C. Ba/F3-parental cells were grown in the presence of 10 ng/ml IL-3. NRAS-G12D or NRAS-G12V transformed Ba/F3 cell lines (Ba/F3-NRAS-G12D, and Ba/F3-NRAS-G12V, respectively) were cultured without IL-3, which were established by the procedure described as previously (30).

#### 2.2.2 Anti-Proliferation Assay

Approximately 3.0 × 10^3^ cells/well were seeded in a 96-well cell culture plate. After stabilization, 1/3 serially diluted compound in DMSO was treated to the cell. After 72 h incubation at 37°C, the cell viability was assessed with CellTiter Glo (Promega, G7572) GI_50_ values were measured by Graphpad prism 6.0 software. All GI_50_ values were obtained in duplicate with three independent assays and averages with standard deviation are presented.

#### 2.2.3 Western Blot

Approximately 1 × 10^6^ cells per well were seeded in a 6-well cell culture plate. After cell adhesion, each compounds were treated for 2 h with indicated concentration and conducted brief washing twice by ice-cold PBS. Cells were lysed by NP40 buffer (50 mM Tris–HCl pH 7.4, 1% NP40, 2 mM EDTA, 150 mM NaCl) in the presence of protease inhibitor cocktail (Roche, #11873580001) and phosphatase inhibitor cocktail (Roche, #04906837001). Each sample was loaded with same amount of total protein and separated by SDS-PAGE gel. After transferred to nitrocellulose membrane, blocking with 5% skim milk (in TBS/T) was followed. The membrane was incubated 4°C for overnight with uniformly diluted primary antibodies at 1:1,000 (v/v) in TBS/T. The following primary antibodies were used: anti-p-AKT (Cell Signaling, #9271), anti-p-ERK1/2 (Cell Signaling, #4370), anti-PARP (Cell Signaling, #9542), anti-GAPDH (Cell Signaling, #5174), anti-p-70S6K1 (Cell Signaling, #9204), anti-p-JNK(Cell Signaling, #9251), anti-p-p38 (Cell Signaling, #9216), and anti-p-MEK1/2 (Santa Cruz, sc-81503). Each membrane was incubated with HRP-conjugated goat anti-Rabbit secondary antibody (Gendepot, SA002-500) or HRP-conjugated goat anti-Mouse secondary antibody (Gendepot, SA001-500) for 1 h (1:10,000, v/v) in TBS/T at room temperature and followed ECL solution treatment. Chemiluminescence signals were detected by ImageQuant™ LAS 4000 (GE Healthcare).

#### 2.2.4 Flow Cytometry Analysis

For apoptosis analysis, cells (1 × 10^6^ cells per sample) were incubated with each compounds for 24 h. Cells were subjected to brief ice-cold PBS washing and subjected to staining with Alexafluor488-conjugated annexin V (Thermo Fisher, A13201), and propidium iodide (PI, Thermo Fisher, P3560). Thereafter, apoptotic cells were classified by FACS Accuri™ C6 Plus (BD Biosciences). Debris and unstained cells and were excluded by gating.

For cell cycle analysis, cells (1 × 10^6^ cells per sample) were incubated with each compounds for 24 h. Cells were subjected to brief ice-cold PBS washing and fixed with 70% EtOH for 1 h at −20°C. Cells were stained with PI/RNase staining solution (Cell signaling, #4087) and incubated 30 min at room temperature in a dark condition. Stained cells were subjected to flow cytometry analysis. Debris and unstained cells and were excluded by gating.

#### 2.2.5 Migration and Invasion Assay

For migration assay, scratch assay was conducted. Each cells (2.0 × 10^5^ cells/well) were seeded in 24-well plates. After cellular attachment, cells were scratched with a SPLScarTM Scratcher (SPL Life Sciences, #201924) and the detached cells were removed by PBS washing twice. Cells were incubated in complete media with 0.01 μM concentrations of each compounds for indicated time. The images were acquired before and after incubation with compounds (40× magnification), and percent of migration were accessed using ImageJ (n = 3).

For invasion assay, Boyden chamber assay carried out. Transwell insert was precoated with matrigel (Corning, #354248). Cells were seeded in the transwell chamber insert (8 μm pore size) at a density of 5.0 × 10^5^ cells/well after serum starvation for overnight. The cells were incubated with 0.01 μM concentration of each compounds of for 48 h at 37°C. The non-invaded cells were eliminated. Crystal violet solution was used to staining the invaded cells. Cells were observed and photographed with 100× magnification. Stained cells were automatically counted and quantified (n = 3).

#### 2.2.6 Colony Formation Assay

Colony formation assay was conducted for 2D clonogenic assay. Approximately 1 × 10^3^ cells/well were seeded in 6-well plate. After cellular attachment, indicated concentrations of compounds were treated for 14 days at 37°C. Colonies were stained by crystal violet solution for 30 min. The total area of each well was observed without magnification, and the number of colonies of each well were accessed using ImageJ software (n = 3).

Soft agar assay was conducted for 3D clonogenic assay. On the 0.7% bottom agar, cells were seeded in 6-well plate (5 × 10^3^ cells/well) with 0.35% low melting agar (Lonza, #50101) containing the complete media. The cells were treated with indicated compounds for 14 days at 37°C. Colonies were stained by iodonitrotetrazolium chloride (Sigma-Aldrich, I8377) for overnight. The total area of each well was observed without magnification, and the number of colonies of each well were determined using ImageJ software (n = 3).

## 3 Results

### 3.1 Anti-Proliferative Activities of Fourteen GNF-7 Derivatives on Ba/F3 Cells Transformed With mtRAS (NRAS-G12D and NRAS-G12V) and Ba/F3 Parental Cells

To evaluate anti-proliferative activities of fourteen GNF-7 derivatives, Ba/F3 cells transformed with NRAS-G12D and NRAS-G12V were utilized (**Table 1**). We investigated the effects of various amine substituents on the left part of GNF-7 structure (compounds 1–9). We found that derivatives 2–4 and SIJ1795 resulted in similar to 5-fold increased anti-proliferative activities (GI_50_s = 0.075 to 0.230 µM) on Ba/F3-NRAS-G12D and Ba/F3-NRAS-G12V compared with GNF-7. Among these five derivatives, SIJ1795 exhibited the highest potencies (GI_50_ = 0.082 µM on Ba/F3-NRAS-G12D, GI_50_ = 0.075 µM on Ba/F3-NRAS-G12V). We further modified the left part of GNF-7 structure by installing 5-membered pyrazole moieties (compounds 10–13 and SIJ1772) and made a notable observation that the introduction of pyrazole moieties resulted in increased anti-proliferative activities against Ba/F3-NRAS-G12D (GI_50_s = 0.077 to 0.193 µM) and Ba/F3-NRAS-G12V (GI_50_s = 0.096 to 0.280 µM) compared with those of GNF-7. Among the five pyrazole-based derivatives explored, SIJ1772 displayed the highest potencies (GI_50_ = 0.077 µM on Ba/F3-NRAS-G12D, GI_50_ = 0.096 µM on Ba/F3-NRAS-G12V). Taken together, SIJ1795 and SIJ1772, novel and potent GNF-7 derivatives, showed 3 to 5-fold enhanced anti-proliferative activities relative to those of GNF-7 and exhibited two-digit nanomolar potencies against both Ba/F3-NRAS-G12D (GI_50_s = 0.077 to 0.082 µM) and Ba/F3-NRAS-G12V (GI_50_s = 0.075 to 0.096 µM) cells. Those derivatives displayed 20 to 27-fold differential cytotoxicity against both Ba/F3-NRAS-G12D and Ba/F3-NRAS-G12V cells relative to Ba/F3-parental cells. To suggest the target of SIJ1795 and SIJ1772 on the cells with mtNRAS, we conducted biochemical kinase assay and western blot (**Figure S1**). The results of biochemical kinase assay reveal that SIJ1795 and SIJ1772 possess 2 to 3-fold enhanced inhibitory activities compared to GNF-7. Moreover, the results of western blot show that the phosphorylation of 70S6K1 (downstream molecule of ACK), JNK, p38 (downstream molecule of GCK) were effectively inhibited by SIJ1795 and SIJ1777. Therefore, we believe that synthetic lethality of ACK1 and GCK would be the target of SIJ1795 and SIJ1772. Considering anti-proliferative activities against Ba/F3-NRAS-G12D and Ba/F3-NRAS-G12V, we selected SIJ1795 and SIJ1772 as representative compounds for further biological evaluation.

**Table 1:** Anti-proliferative activities on Ba/F3-NRAS-G12D, Ba/F3-NRAS-G12V, and Ba/F3-parental cells.

Entry	GI_50_ (µM)*^a^*		
	Ba/F3	Ba/F3	Ba/F3
	NRAS-G12D	NRAS-G12V	Parental
GNF-7	0.250 ± 0.026	0.396 ± 0.036	0.923 ± 0.059
**1**	0.586 ± 0.091	0.928 ± 0. 132	>50
**2**	0.116 ± 0.037	0.181 ± 0.042	0.827 ± 0.142
**3**	0.120 ± 0.039	0.134 ± 0.045	1.059 ± 0.055
**4**	0.230 ± 0.129	0.213 ± 0.053	0.857 ± 0.108
**5**	0.457 ± 0.141	0.351 ± 0.034	5.730 ± 0.148
**6**	0.569 ± 0.028	0.428 ± 0.161	1.474 ± 0.317
**7**	0.588 ± 0.072	0.429 ± 0.080	3.779 ± 1.890
**8**	1.255 ± 0.006	0.944 ± 0.112	3.919 ± 0.722
**SIJ1795** (**9**)	0.082 ± 0.031	0.075 ± 0.045	2.022 ± 0.419
**10**	0.193 ± 0.007	0.280 ± 0.079	0.630 ± 0.104
**11**	0.185 ± 0.015	0.259 ± 0.126	1.164 ± 0.330
**12**	0.122 ± 0.001	0.216 ± 0.005	1.156 ± 0.424
**13**	0.114 ± 0.018	0.130 ± 0.028	0.889 ± 0.061
**SIJ1772** (**14**)	0.077 ± 0.016	0.096 ± 0.009	1.924 ± 0.086

^a^GI_50_ represents the concentration at which a compound causes half-maximal growth inhibition. Ba/F3-NRAS-G12D, Ba/F3-NRAS-G12V, and Ba/F3-parental cells were treated with inhibitors for 72 h. Average GI_50_ values with SD (n = 3, duplicate) are shown.

### 3.2 Anti-Proliferative Activities of the Fourteen GNF-7 Derivatives Against Cancer Cells Harboring mtRAS

Encouraged by fourteen GNF-7 derivatives capable of suppressing the proliferation against Ba/F3 cells transformed with NRAS-G12D and NRAS-G12V, we assessed anti-proliferative activities of GNF-7 and its derivatives against various cancer cells harboring mtRAS such as H358 (KRAS-G12C), AsPC-1 (KRAS-G12D), DU-145 (KRAS-G12V), SW480 (KRAS-G12V), MDA-MB-231 (KRAS-G13D), HT-29 (KRAS-Q61L), and OCI-AML3 (NRAS-Q61L) as shown in **Table 2**. GNF-7 effectively suppressed proliferation of cancer cells harboring mtRAS with submicromolar potencies (GI_50_s = 0.169 to 0.938 µM). In accordance with the observation form anti-proliferative activities against Ba/F3 cells transformed with mtRAS (NRAS-G12D and NRAS-G12V), derivatives with modified substituents (compounds 2–4 and SIJ1795) showed 2 to 11-fold increased anti-proliferative activities against all cancer cells harboring mtRAS compared with GNF-7. Moreover, derivatives containing 5-membered pyrazole moiety (10–14) displayed similar to 10-fold increased anti-proliferative potencies (10–14, GI_50_s = 0.029 to 0.532 µM). Moreover, all fourteen compounds exhibited more than 8-fold differential cytotoxicity against OCI-AML3 (NRAS-Q61L) cells relative to U937 (NRAS-wt) cells. It is noteworthy that representative compounds SIJ1795 and SIJ1772 displayed 2 to 10-fold enhanced cellular potencies against all cancer cells harboring mtRAS. (GI_50_s = 0.029 to 0.447 µM). SIJ1795 and SIJ1772 are 64 and 101 times less active on MDA-MB-468 cells harboring wtRAS, respectively than on SW480 cells with KRAS-G12V mutation. In addition, antiproliferative activity of SIJ1795 and SIJ1772 is 476- and 1182-fold lower on MDA-MB-468 cells, respectively than on HCT-116 harboring KRAS-G13D mutation. Therefore, both SIJ1795 and SIJ1772 are capable of inhibiting more strongly cancer cells harboring mtRAS than wtRAS-expressing cancer cells (MDA-MB-468 and U937). The results suggest that SIJ1795 and SIJ1772 are capable of strongly suppressing mtRAS signaling pathway.

**Table 2:** Anti-proliferative activities of the fourteen GNF-7 derivatives on cancer cells harboring mtRAS (H358, AsPC-1, DU-145, SW480, HCT-116, MDA-MB-231, HT-29, OCI-AML3) and KRAS, NRAS-wt (U937 and MDA-MB-468) cells.

Entry	GI_50_ (µM)*^a^*									
	H358 (KRAS-G12C)	AsPC-1 (KRAS-G12D)	DU-145 (KRAS-G12V)	SW480 (KRAS-G12V)	HCT-116 (KRAS-G13D)	MDA-MB-231 (KRAS-G13D)	HT-29 (KRAS-Q61L)	OCI-AML3 (NRAS-Q61L)	MDA-MB-468 (KRAS, NRAS-wt)	U937 (KRAS, NRAS-wt)
GNF-7	0.922 ± 0.093	0.448 ± 0.027	0.288 ± 0.057	0.760 ± 0.063	0.169 ± 0.061	0.296 ± 0.039	0.938 ± 0.034	0.229 ± 0.010	>50	4.775 ± 0.926
**1**	1.384 ± 0.096	2.019 ± 0.056	1.042 ± 0.400	4.511 ± 0.873	1.237 ± 0.554	1.115 ± 0.290	1.307 ± 0.054	0.611 ± 0.065	>50	>50
**2**	0.201 ± 0.023	0.257 ± 0.129	0.040 ± 0.020	0.293 ± 0.050	0.015 ± 0.009	0.090 ± 0.000	0.143 ± 0.010	0.101 ± 0.022	20.180 ± 8.771	1.694 ± 0.624
**3**	0.265 ± 0.004	0.136 ± 0.011	0.030 ± 0.008	0.382 ± 0.052	0.018 ± 0.006	0.049 ± 0.012	0.227 ± 0.012	0.080 ± 0.016	>50	2.225 ± 0.163
**4**	0.236 ± 0.029	0.135 ± 0.018	0.032 ± 0.014	0.163 ± 0.000	0.025 ± 0.010	0.083 ± 0.022	0.126 ± 0.002	0.044 ± 0.014	26.060 ± 7.900	1.044 ± 0.225
**5**	5.143 ± 0.368	4.165 ± 2.168	1.878 ± 0.125	6.445 ± 0.264	2.541 ± 1.814	1.111 ± 0.127	3.122 ± 0.460	0.538 ± 0.162	>50	4.725 ± 0.488
**6**	2.055 ± 1.133	3.170 ± 0.244	1.170 ± 0.124	2.054 ± 0.051	1.529 ± 0.390	0.646 ± 0.068	3.253 ± 0.176	0.374 ± 0.024	>50	2.870 ± 0.142
**7**	0.871 ± 0.141	1.676 ± 0.589	0.388 ± 0.045	1.329 ± 0.005	0.480 ± 0.275	0.620 ± 0.153	0.733 ± 0.001	0.606 ± 0.068	>50	17.396 ± 4.220
**8**	8.980 ± 0.246	6.652 ± 0.102	3.986 ± 0.566	5.794 ± 0.657	5.808 ± 0.965	2.793 ± 0.658	2.145 ± 0.109	0.687 ± 0.154	>50	6.064 ± 2.181
**SIJ1795** (**9**)	0.203 ± 0.012	0.144 ± 0.023	0.077 ± 0.043	0.245 ± 0.012	0.033 ± 0.006	0.053 ± 0.008	0.154 ± 0.042	0.052 ± 0.052	15.725 ± 5.310	1.004 ± 0.126
**10**	0.130 ± 0.009	0.105 ± 0.034	0.051 ± 0.046	0.275 ± 0.029	0.068 ± 0.028	0.050 ± 0.012	0.366 ± 0.152	0.059 ± 0.007	>50	2.702 ± 0.668
**11**	0.240 ± 0.042	0.318 ± 0.010	0.089 ± 0.047	0.286 ± 0.039	0.045 ± 0.016	0.108 ± 0.026	0.355 ± 0.054	0.103 ± 0.001	>50	7.004 ± 1.117
**12**	0.106 ± 0.012	0.275 ± 0.047	0.034 ± 0.025	0.532 ± 0.150	0.066 ± 0.055	0.071 ± 0.018	0.131 ± 0.004	0.046 ± 0.016	>50	0.934 ± 0.057
**13**	0.147 ± 0.031	0.186 ± 0.022	0.076 ± 0.026	0.269 ± 0.061	0.077 ± 0.027	0.055 ± 0.020	0.164 ± 0.002	0.036 ± 0.007	16.158 ± 6.021	0.690 ± 0.035
**SIJ1772** (**14**)	0.125 ± 0.017	0.170 ± 0.032	0.029 ± 0.003	0.447 ± 0.173	0.038 ± 0.028	0.058 ± 0.001	0.192 ± 0.094	0.065 ± 0.029	44.933 ± 6.255	9.592 ± 0.832

^a^GI_50_ represents the concentration at which a compound causes half-maximal growth inhibition. Indicated cells were treated with inhibitors for 72 h. Average GI_50_ values with SD (n = 3, duplicate) are shown.

### 3.3 Signaling Inhibitory Activities on Ba/F3 Cells Transformed With mtRAS (NRAS-G12D, NRAS-G12V) and Cancer Cells Harboring mtRAS

**Figure 1:**
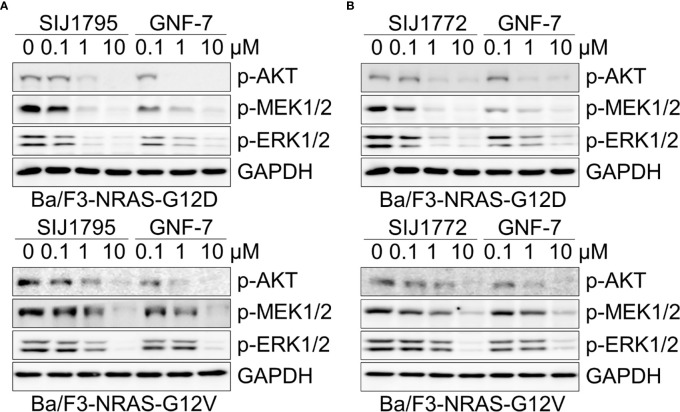
The effects of **(A)** SIJ1795 and **(B)** SIJ1772 on MAPK and PI3K/AKT signaling pathways in Ba/F3 cells transformed with mtRAS (NRAS-G12D and NRAS-G12V). Cells were treated with 0.1, 1, and 10 μM of SIJ1795, SIJ1772, and GNF-7 for 2 h. Western blot analysis was conducted to evaluate the phospho-AKT, -MEK, -ERK levels. GAPDH was used as the loading control.

**Figure 2:**
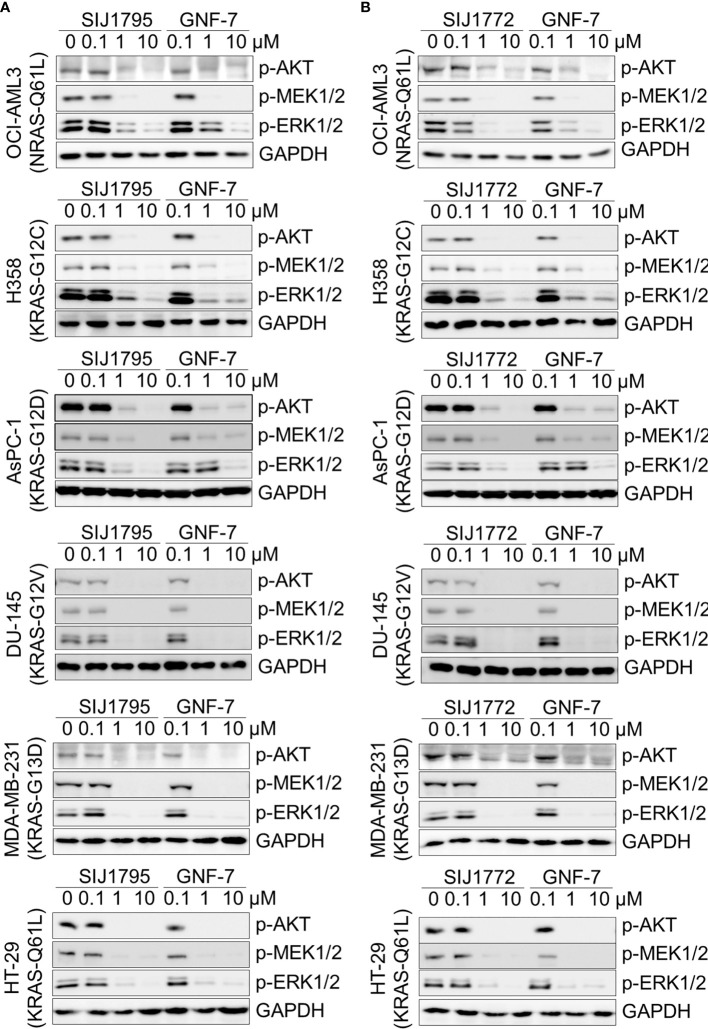
The effect of **(A)** SIJ1795 and **(B)** SIJ1772 on mtRAS downstream signaling pathways (MAPK, PI3K/AKT signaling) in cancer cells harboring mtRAS. Cells were treated with 0.1, 1, and 10 μM of SIJ1795, SIJ1772, and GNF-7 for 2 h. Western blot analysis was conducted to evaluate the phospho-AKT, -MEK, -ERK levels. GAPDH was used as the loading control.

Oncogenic RAS mutation stimulates diverse downstream cellular signaling pathways, including MAPK and PI3K/AKT pathway (31). To investigate correlation between the anti-proliferative activities of the GNF-7 derivatives and mtRAS downstream signaling inhibition, we assessed Western blot analysis using Ba/F3-NRAS-G12D, Ba/F3-NRAS-G12V, and mtRAS-expressing cancer cells. As shown in **Figure 1**, both SIJ1795 and SIJ1772 inhibited the activation of MEK, ERK, and AKT on Ba/F3-NRAS-G12D and Ba/F3-NRAS-G12V cells in a concentration-dependent manner. Both substances completely inhibited phospho-AKT, -MEK, -ERK levels at 10 µM in Ba/F3 cells transformed with mtRAS (NRAS-G12D and NRAS-G12V). Inhibitory capabilities of SIJ1772 and SIJ1795 on phosphorylation level of MEK, ERK, and AKT at 1 µM concentration were shown to be slightly increased relative to that of GNF-7, which is in agreement with anti-proliferative activities on Ba/F3-NRAS-G12D and Ba/F3-NRAS-G12V cells. Moreover, in cancer cells with mtRAS, signaling inhibitory activities of SIJ1795 or SIJ1772 are comparable to those of GNF-7 (**Figure 2**). Taken together, SIJ1795 and SIJ1772 block mtRAS downstream signaling (MAPK and PI3K/AKT pathways) in not only Ba/F3 cells transformed with mtRAS (NRAS-G12D and NRAS-G12V), but also cancer cells expressing mtRAS in a concentration dependent fashion.

### 3.4 Effects of SIJ1795 and SIJ1772 on Apoptosis Induction and Cell Cycle Arrest in Ba/F3 Cells Transformed With mtRAS (NRAS-G12D and NRAS-G12V) and Cancer Cells Harboring mtRAS

**Figure 3:**
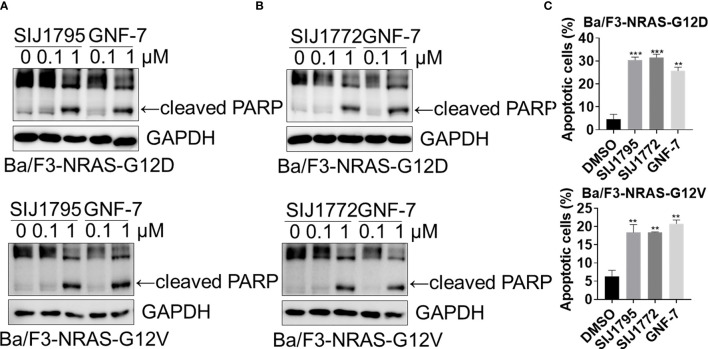
**(A, B)** The effects of SIJ1795 and SIJ1772 on apoptosis induction in Ba/F3 cells transformed with mtRAS (NRAS-G12D and NRAS-G12V). Cells were treated with 0.1 and 1 μM of SIJ1795, SIJ1772, and GNF-7 for 24 h Western blot analysis was conducted to evaluate the cleaved PARP and level. GAPDH was used as a loading control. **(C)** Apoptotic cell population (annexin V-positive) was determined by flow cytometry analysis (n = 3). Cells were treated with 1 μM of indicated compounds for 24 h. Statistical significance was calculated by a one-way ANOVA analysis (**p < 0.01, ***p < 0.001).

**Figure 4:**
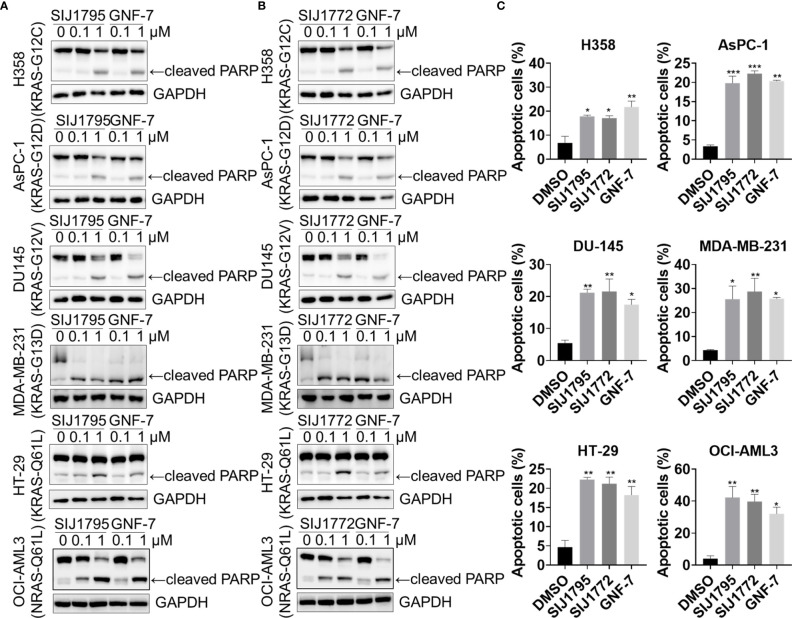
The effects of **(A)** SIJ1795 and **(B)** SIJ1772 on apoptosis induction in cancer cells harboring mtRAS. Cells were treated with 0.1 and 1 μM of SIJ1795 SIJ1772, and GNF-7 for 24 h. Western blot analysis was conducted to evaluate the cleaved PARP and level. GAPDH was used as a loading control. **(C)** Apoptotic cell population (annexin V-positive) was determined by flow cytometry analysis (n = 3). Cells were treated with 1 μM of indicated compounds for 24 h. Statistical significance was determined using a one-way ANOVA analysis (*p < 0.05, **p < 0.01, ***p < 0.001).

**Figure 5:**
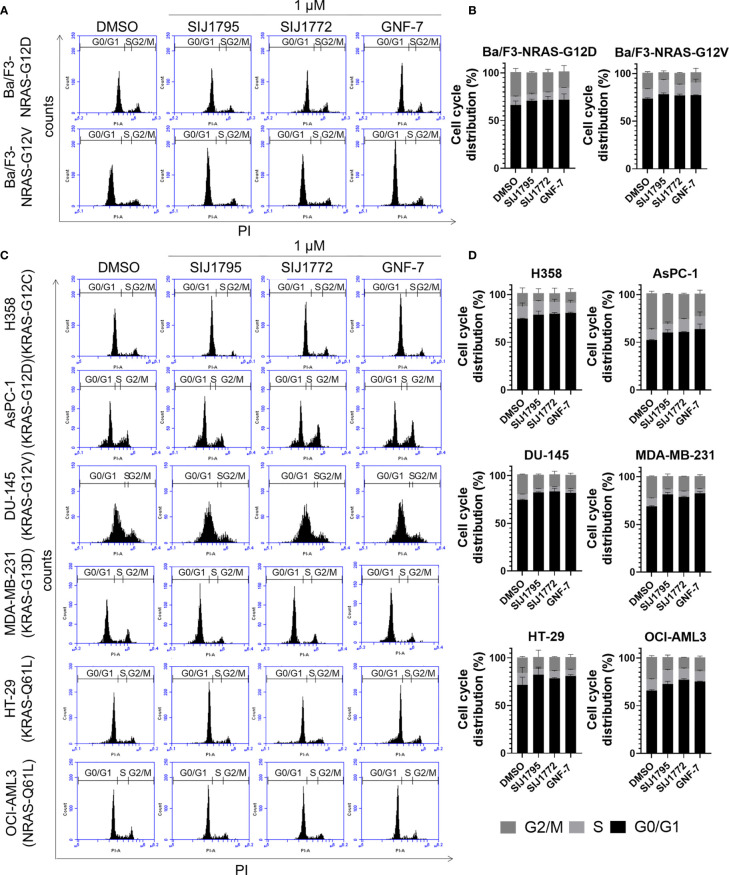
The effects of SIJ1795 and SIJ1772 on cell cycle arrest against in **(A, B)** Ba/F3 cells transformed with mtRAS (NRAS-G12D and NRAS-G12V) and **(C, D)** cancer cells harboring mtRAS. Cells were treated with 1 µM of SIJ1795, SIJ1772, and GNF-7 for 24 h. Cells were stained by PI and analyzed the percentage of cell cycle phase by flow cytometry.

We performed Western blot and flow cytometry analysis to evaluate the effects of the representative derivatives on apoptosis or cell cycle arrest induction. First, we measured cleaved PARP level, a well-known pro-apoptotic marker It is worthwhile to note that SIJ1795 and SIJ1772 increased cleaved PARP level against Ba/F3-NRAS-G12D and Ba/F3-NRAS-G12V cells at 1 μM concentration (**Figures 3A, B**). In addition, the level of cleaved PARP was notably increased at 1 μM concentration of SIJ1795 or SIJ1772 in the cancer cells harboring mtRAS (**Figures 4A, B**). We next carried out flow cytometry analysis and evaluated apoptotic cells population using annexin V/propidium iodide (PI) staining. Treatment of SIJ1795 and SIJ1772 noticeably led to an increase of apoptotic cells in Ba/F3-NRAS-G12D and Ba/F3-NRAS-G12V cells, which is consistent with the results of Western blot analysis (**Figure 3C**, **Supplementary Figure S2**). Moreover, SIJ1795 and SIJ1772 induced apoptosis against cancer cells harboring mtRAS (**Figure 4C**, **Supplementary Figure S3**). Apoptosis induction capabilities of SIJ1795 or SIJ1772 are comparable to that of GNF-7. We then analyzed cell cycle by flow cytometry analysis and observed that G0/G1 arrest was induced by SIJ1795 or SIJ1772 at 1 µM, (**Figure 5**) which correlates with our previous findings (27). Taken together, our results demonstrate that SIJ1795 and SIJ1772 are capable of inducing apoptosis and G0/G1 cell cycle arrest on Ba/F3 cells transformed with mtRAS (NRAS-G12D and NRAS-G12V), and cancer cells harboring mtRAS.

### 3.5 Migration and Invasion Inhibitory Activity of SIJ1795 and SIJ1772 on Cancer Cells Harboring mtRAS

**Figure 6:**
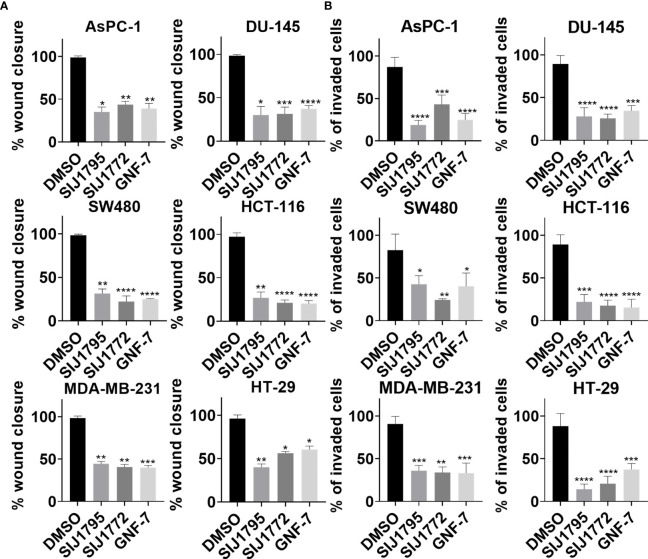
The effects of SIJ1795 and SIJ1772 on migration and invasion activities of cancer cells harboring mtRAS. **(A)** Scratch assay result for cell migration ability determination. Each cell monolayers were scratched and incubated with test compounds at 0.01 μM. After 8 h (MDA-MB-231) or 24 h (DU-145, SW480, HCT-116) or 48 h (AsPC-1) or 72 h (HT-29), cells were stained with crystal violet. Migrated areas were photographed with 40× magnification. Migrated area was determined by ImageJ (n = 3). **(B)** Boyden chamber assay result for cell invasion ability determination. Indicated compounds of 0.01 μM were treated and incubated for 48 h Invaded cells were stained by crystal violet and photographed. Stained cells were counted by ImageJ (n = 3). Statistical significance was determined using a one-way ANOVA analysis (*p < 0.05, **p < 0.01, ***p < 0.001, ****p < 0.0001).

Previous studies have shown that KRAS activating mutation is associated with enhanced cell migration in invasion activities in many types of cancer, such as pancreatic ductal adenocarcinoma (32), prostate cancer (33), colon cancer (34, 35), and breast cancer (36). Accordingly, we investigated inhibitory capabilities of SIJ1795 and SIJ1772 on migration and invasion against cancer cells with mtRAS (**Figure 6**, **Supplementary Figures S4, S5**). It is noteworthy that 0.01 μM of SIJ1795 potently suppressed migration and invasion capabilities of cancer cells harboring mtRAS, regardless their mutation status. Furthermore, in this investigation, we noticed over 50% reduction in migration and invasion capabilities of cancer cells with mtRAS including H358, AsPC-1, DU-145, SW480, and MDA-MB-231 when treated with both SIJ1795 and SIJ1772 at 0.01 µM.

### 3.6 Colony Formation Inhibitory Effect of SIJ1795 and SIJ1772 on Cancer Cells Harboring mtRAS

**Figure 7:**
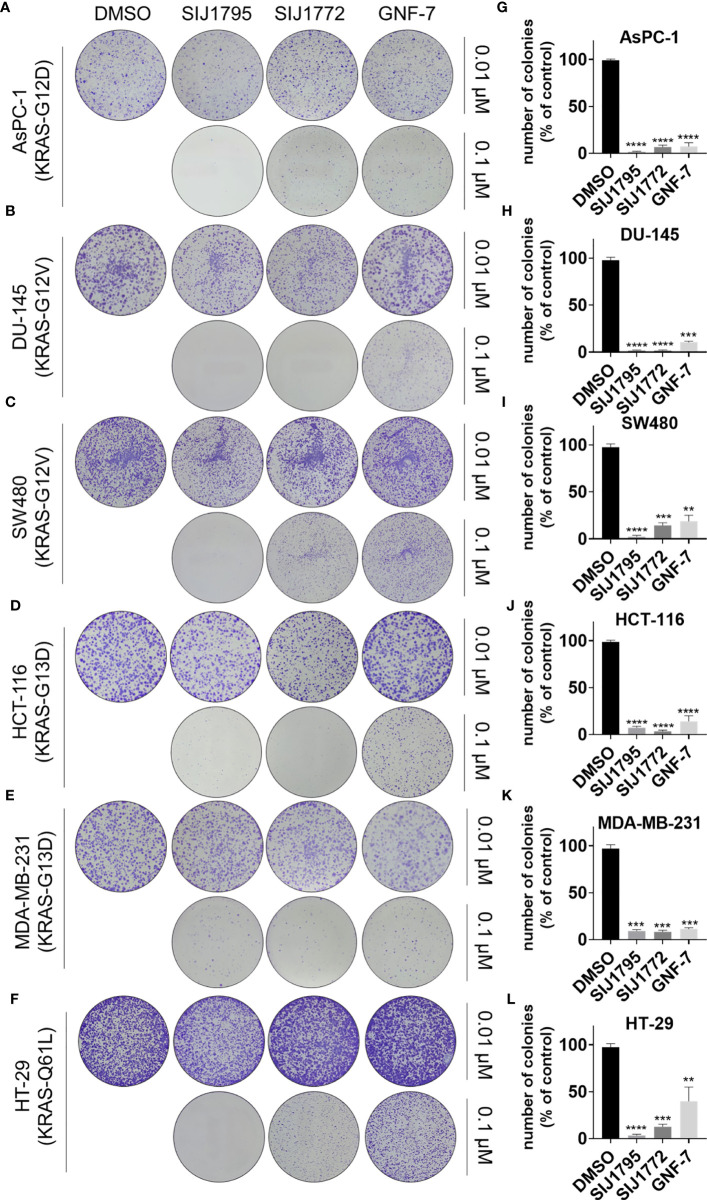
**(A–F)** Colony formation assay results evaluating the effects of SIJ1795 and SIJ1772 on clonogenic abilities of on cancer cells harboring mtRAS. Cells were incubated with indicated compounds (0.01 µM or 0.1 µM) for 14 days. After staining with crystal violet, colonies were photographed without magnification. **(G–L)** Number of colonies (% of control) upon treatment with indicated compounds at 0.1 µM concentration were determined by ImageJ (n = 3, respectively). Statistical significance was calculated by using a one-way ANOVA analysis (**p < 0.01, ***p < 0.001, ****p < 0.0001).

**Figure 8:**
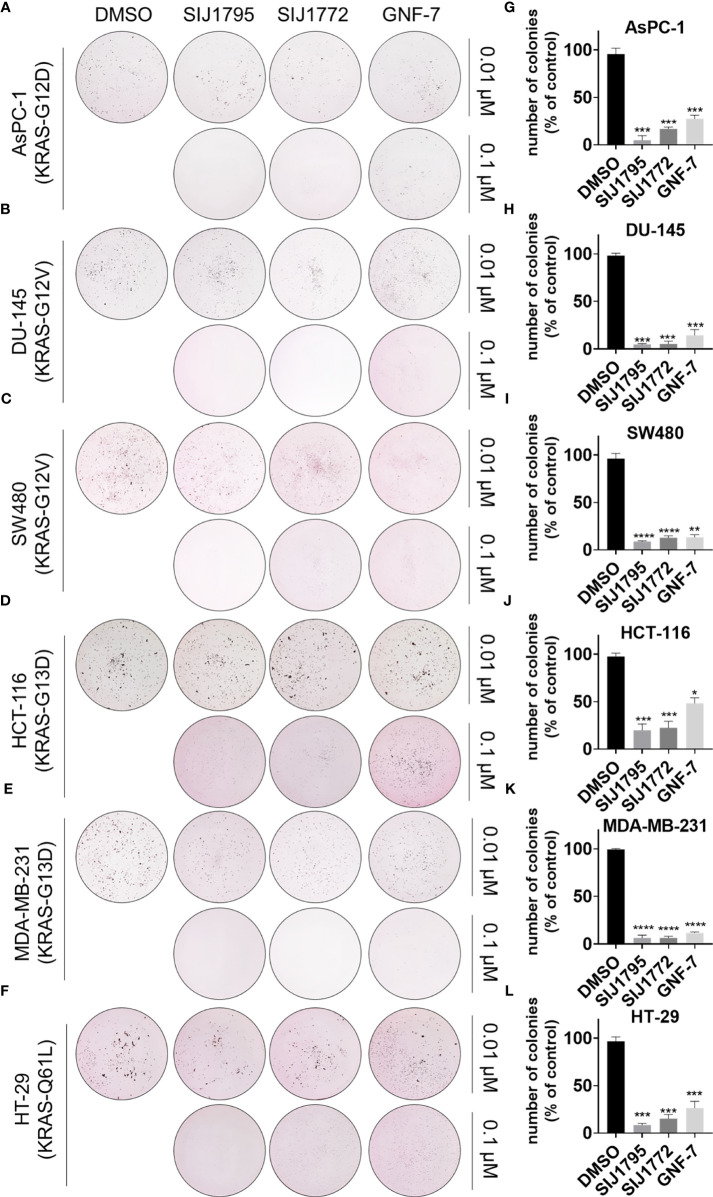
**(A–F)** Soft agar assay results for evaluating the effect of SIJ1795 and SIJ1772 on anchorage independent growth abilities on cancer cells harboring mtRAS. Cells were incubated with indicated compounds (0.01 µM or 0.1 µM) for 14 days. After staining with crystal violet, colonies were photographed without magnification. **(G–L)** Number of colonies (% of control) upon treatment with indicated compounds at 0.1 µM concentration were determined by ImageJ (n = 3, respectively). Statistical significance was calculated by a one-way ANOVA analysis (*p < 0.05, **p < 0.01, ***p < 0.001, ****p < 0.0001).

Subsequently, we examined tumorigenesis inhibitory effects of SIJ1795 and SIJ1772 on cancer cells harboring mtRAS by conducting 2D and 3D clonogenic assay (colony formation assay and soft agar assay, respectively). As shown in **Figures 7**, **8**, SIJ1795 and SIJ1772 suppressed colony formation and anchorage independent cell growth at 0.1 μM. These results suggest that representative derivatives SIJ1795 and SIJ1772 are capable of significantly inhibiting tumorigenesis of cancer cells harboring mtRAS.

## 4 Discussion

Blocking mtRAS downstream signaling pathway can be an effective approach to discover novel inhibitors targeting mtRAS. In our previous study (25, 27), we demonstrated that dual inhibition of ACK1 and GCK by multi-targeted kinase, GNF-7 and its derivatives, displays strong capabilities of cellular inhibition on both Ba/F3 cells transformed with NRAS-G12D and OCI-AML3, a human cancer cell harboring NRAS-Q61L mutation. We also observed that these anti-proliferative effects exerted by GNF-7 and its derivatives are accompanied by substantial induction of apoptosis and G0/G1 arrest through suppressing AKT/mTOR signaling pathway and downstream molecules of GCK in Ba/F3-NRAS-G12D and OCI-AML3 cells. In addition, we previously reported (28, 29) that GNF-7 and its derivatives as pan-class (class I/II/III) BRAF inhibitors and described that the use of multi-targeted kinase inhibitors may be an useful tactic to block mtRAS signaling pathway. Considering all these preceding findings, we attempted to expand the application of GNF-7 derivatives as a means of a promising anticancer strategy targeting mtRAS *via* blocking the downstream signaling pathway.

As background for the exploration described above, we made significant observations that GNF-7 derivatives display two-digit nanomolar potencies against Ba/F3s transformed with mtRAS (NRAS-G12D and NRAS-G12V). Particularly, SIJ1795 and SIJ1772 exhibited 3 to 5-fold increased activities against Ba/F3-NRAS-G12D and Ba/F3-NRAS-G12V compared with GNF-7 (GI_50_s = 0.075 to 0.096 µM). In agreement with the anti-proliferative activities, Western blot analysis shows that both SIJ1795 and SIJ1772 completely attenuate the level of phospho-MEK and -ERK at 10 µM. This indicates that SIJ1795 and SIJ1772 exert anti-proliferative activities against Ba/F3 cells transformed with NRAS-G12D and NRAS-G12V by effectively blocking oncogenic RAS signaling pathway.

To expand the applicability of GNF-7 derivatives on mtRAS cancer cells, we subsequently evaluated fourteen GNF-7 derivatives against cancer cells harboring mtRAS: H358 (KRAS-G12C), AsPC-1 (KRAS-G12D), DU-145 (KRAS-G12V), SW480 (KRAS-G12V), MDA-MB-231 (KRAS-G13D), HT-29 (KRAS-Q61L), and OCI-AML3 (NRAS-Q61L). In accordance with prior expectations, anti-proliferation activities for this series of derivatives are well translated to various cancer cells with mtRAS. It is of worth to mention that differential cytotoxicity of all fourteen derivatives is enhanced by 8-fold on OCI-AML3 (NRAS-Q61L) cells in relative to U937 (NRAS-wt) cells. Notably, in this investigation, we observed that the representative derivatives, SIJ1795 and SIJ1772 exhibit 2 to 10-fold enhanced cellular potencies against all cancer cells harboring mtRAS (GI_50_s = 0.029 to 0.447 µM) compared to GNF-7, which is also indicative of strong capabilities of SIJ1795 and SIJ1772 on blocking mtRAS signaling pathway. Besides, SIJ1795 and SIJ1772 effectively suppressed phosphorylation level of AKT, MEK, and ERK at 1 µM, and induced apoptosis as well as G0/G1 arrest, contributing to their observed anti-proliferative activities against cancer cells with mtRAS. It is worth recalling that both SIJ1795 and SIJ1772 are multi-targeted kinase inhibitors and anti-proliferative effects of multi-targeted inhibitors are not fully dependent on the on-target signaling inhibitory effect. Based on previous studies showing close correlation between enhanced cell migration and RAS activating mutation in various cancers, we successively assessed inhibitory capabilities of SIJ1795 and SIJ1772 against cancer cells harboring mtRAS on migration, invasion, and anchorage-independent growth. This assessment revealed that SIJ1795 and SIJ1772 at 0.01 µM concentration substantially suppressed migration and invasion capabilities of cancer cells harboring mtRAS including H358, AsPC-1, DU-145, SW480, and MDA-MB-231. Tan et al. showed that bosutinib, which is ACK1 inhibitor, suppressed the mtKRAS NSCLC cell migration and invasion (37). Additionally, SIJ1795 and SIJ1772 significantly inhibit colony formation and anchorage independent cell growth at 0.1 μM.

Taken together, SIJ1795 and SIJ1772 are potent mtRAS signaling blockers and significantly suppress migration and invasion of cancer cells with mtRAS, which suggests that these GNF-7 based derivatives may serve as potential strategy to override mtRAS-driven cancer.

## 5 Conclusion

Targeting mtRAS has been regarded as one of unresolved challenges in oncology drug discovery. Despite intensive efforts to target mtRAS by directly or indirectly inhibiting the protein, AMG-510 (sotorasib) is currently the only FDA-approved drug, which solely targets KRAS-G12C. Accordingly, the development of novel strategies to target mtRAS remains as a great unmet medical need.

In the current effort, we synthesized fourteen derivatives based on GNF-7 and evaluated their anti-proliferative activities against Ba/F3s transformed with mtRAS (NRAS-G12D and NRAS-G12V). We found that nine derivatives strongly suppress the proliferation of those mtRAS Ba/F3 cells. Especially, SIJ1795 and SIJ1772 possess 3 to 5-fold enhanced anti-proliferative activities in Ba/F3-NRAS-G12D and Ba/F3-NRAS-G12V (GI_50_s = 0.075 to 0.096 µM). Moreover, SIJ1975 and SIJ1772 exhibit a higher differential cytotoxicity against Ba/F3-NRAS-G12D and Ba/F3-NRAS-G12V cells (20- to 27-fold) than GNF-7. Notably, Western blot analysis firmly support that SIJ1975 and SIJ1772 suppress phosphorylation of RAS downstream signaling molecules (AKT, MEK, and ERK), indicating both SIJ1975 and SIJ1772 exert anti-proliferative as excellent mtRAS signaling pathway blockers. In addition, we observed that SIJ1975 and SIJ1772 effectively induced apoptosis, G0/G1 arrest, and increase in apoptotic markers in Ba/F3-NRAS-G12D and Ba/F3-NRAS-G12V, which further supports their inhibition capabilities of cellular proliferation against mtRAS Ba/F3 cells.

As part of a study focused on overriding mtRAS, we expanded the application of this series of compounds to various cancer cells harboring KRAS-G12D (AsPC-1), KRAS-G12V (SW480, DU-145), KRAS-G12C (H358), KRAS-G13D (MDA-MB-231), KRAS-Q61L (HT-29), and NRAS-Q61L (OCI-AML3). We noticed that anti-proliferative activities shown in mtRAS Ba/F3 cells are well-translated to various cancer cells harboring mtRAS. Furthermore, it is worth noting that SIJ1975 and SIJ1772 displayed 2 to 10-fold enhanced activities against cancer cells with mtRAS than GNF-7. Both compounds induced apoptosis and G0/G1 arrest as well as they significantly blocked migration, invasion, and anchorage-independent growth in aforementioned cancer cells harboring mtRAS.

On the whole, the combined results of this exploration led us to identification of SIJ1975 and SIJ1772 capable of strongly blocking mtRAS signaling pathway. Thereupon, this study provides fruitful insight into application of multi-targeted kinase inhibitor, GNF-7 and its derivatives, as an effective approach for overriding mtRAS.

## Data Availability Statement

The datasets presented in this study can be found in online repositories. The names of the repository/repositories and accession number(s) can be found in the article/**Supplementary Material**.

## Author Contributions

NK, IS, and YK wrote the manuscript. NK and IS designed experiments. NK, JL, EJe, CL, YN, and SL conducted experiments. IS, EJu, CK, WS, SR, and MK synthesized the GNF-7 derivatives. TS conceived, proofread, and edited the manuscript. All authors contributed to this article and approved the submitted version.

## Funding

This research was supported by the Basic Science Research Program (NRF-2021R1A2C3011992) from the National Research Foundation in Korea.

## Conflict of Interest

The authors declare that the research was conducted in the absence of any commercial or financial relationships that could be construed as a potential conflict of interest.

## Publisher’s Note

All claims expressed in this article are solely those of the authors and do not necessarily represent those of their affiliated organizations, or those of the publisher, the editors and the reviewers. Any product that may be evaluated in this article, or claim that may be made by its manufacturer, is not guaranteed or endorsed by the publisher.

## Supplementary Material

Supplementary Figure 1
**(A)** IC_50_ of SIJ1795, SIJ1772, and GNF-7 against ACK1 and GCK. Each of IC_50_ was acquired by a radiometric biochemical kinase assay. **(B, C)** ACK1 and GCK downstream signaling inhibitory activity of SIJ1795 and SIJ1772 against Ba/F3-NRAS-G12D and OCI-AML cells. Cells were treated with 0.1, 1, 10 μM of SIJ1795, SIJ1772, and GNF-7 for 2 h. Western blot analysis was conducted to evaluate the phospho- 70S6K1, -JNK, -p38 levels. GAPDH was used as the loading control.

Supplementary Figure 2 Apoptosis induction activity of SIJ1795 and SIJ1772 against cancer cells harboring mtRAS. Cells were treated with each compounds of 1 μM for 24 h and subjected to Alexa Flour 488 conjugated annexin V and propidium iodide staining. Flow cytometry analysis were performed.

Supplementary Figure 3 Apoptosis induction activity of SIJ1795 and SIJ1772 cancer cells harboring mtRAS. Cells were treated with each compounds of 1 μM for 24 h and subjected to Alexa Flour 488 conjugated annexin V and propidium iodide staining. Flow cytometry analysis were performed.

Supplementary Figure 4 Migration inhibitory effect of SIJ1795 and SIJ1777 cancer cells harboring mtRAS. Wound healing assay was conducted for cell migration analysis. cell monolayer was scratched and test compounds with 0.01 μM concentration were incubated for indicated time. Cells were stained with crystal violet solution and photographed with 40 × magnification.

Supplementary Figure 5 Invasion inhibitory effect of SIJ1795 and SIJ1777 cancer cells harboring mtRAS. To analyze cell invasion ability, Boyden chamber assay was performed. Wound healing assay was conducted for cell migration analysis. Cell monolayer was scratched and test compounds with 0.01 μM concentration were incubated for indicated time. Cells were stained with crystal violet solution and photographed with 100 × magnification.
