# Supplementary figures and images for: Novel Small Molecules Capable of Blocking mtRAS-Signaling Pathway
Source: Front Oncol. 2021 Dec 9;11:768022. doi: 10.3389/fonc.2021.768022 (PMC8695447; doi:10.3389/fonc.2021.768022)

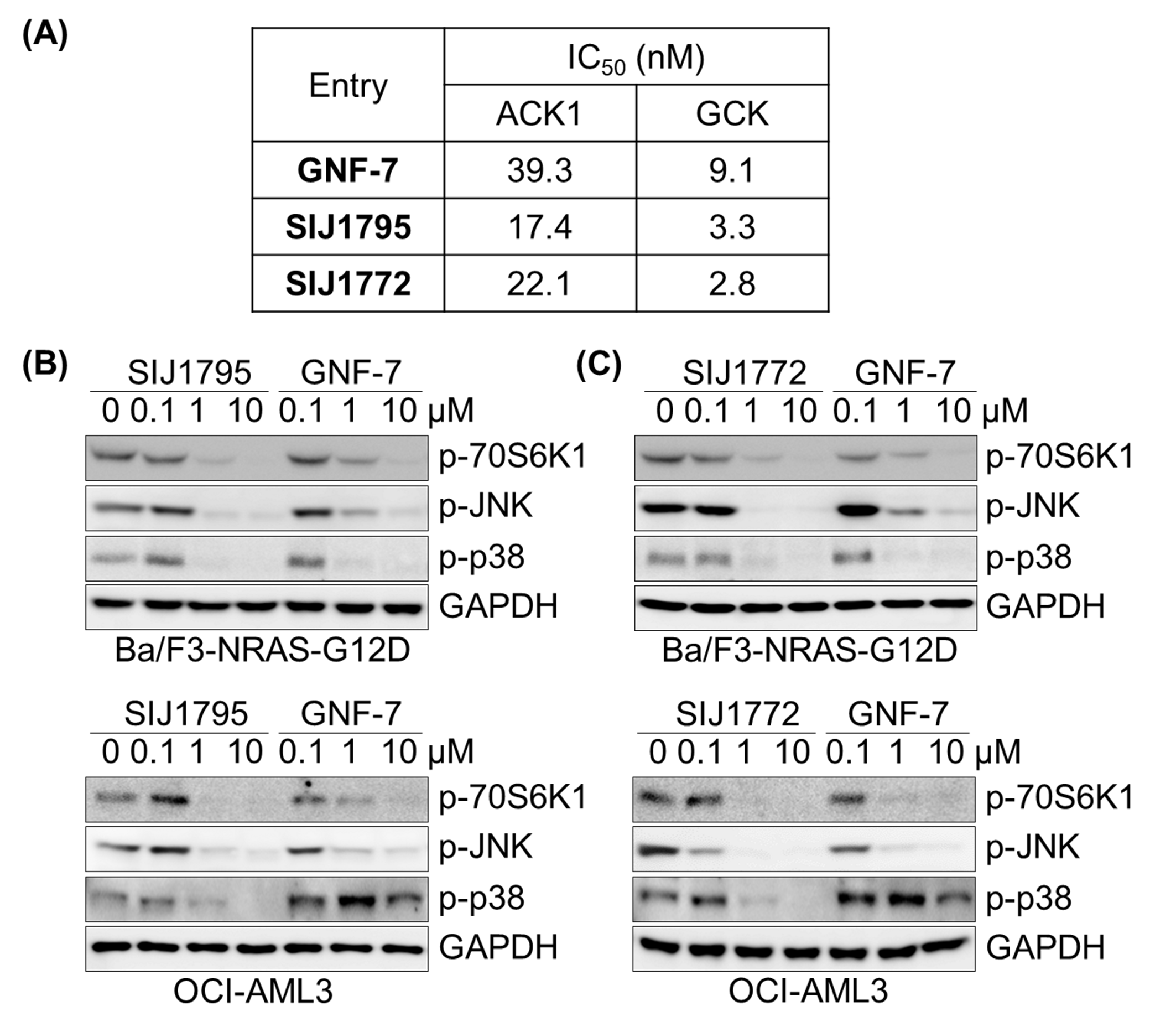

Supplement: Supplementary Figure 1 — (A) IC50 of SIJ1795, SIJ1772, and GNF-7 against ACK1 and GCK. Each of IC50 was acquired by a radiometric biochemical kinase assay. (B, C) ACK1 and GCK downstream signaling inhibitory activity of SIJ1795 and SIJ1772 against Ba/F3-NRAS-G12D and OCI-AML cells. Cells were treated with 0.1, 1, 10 μM of SIJ1795, SIJ1772, and GNF-7 for 2 h. Western blot analysis was conducted to evaluate the phospho- 70S6K1, -JNK, -p38 levels. GAPDH was used as the loading control. [file Image_1.tif]

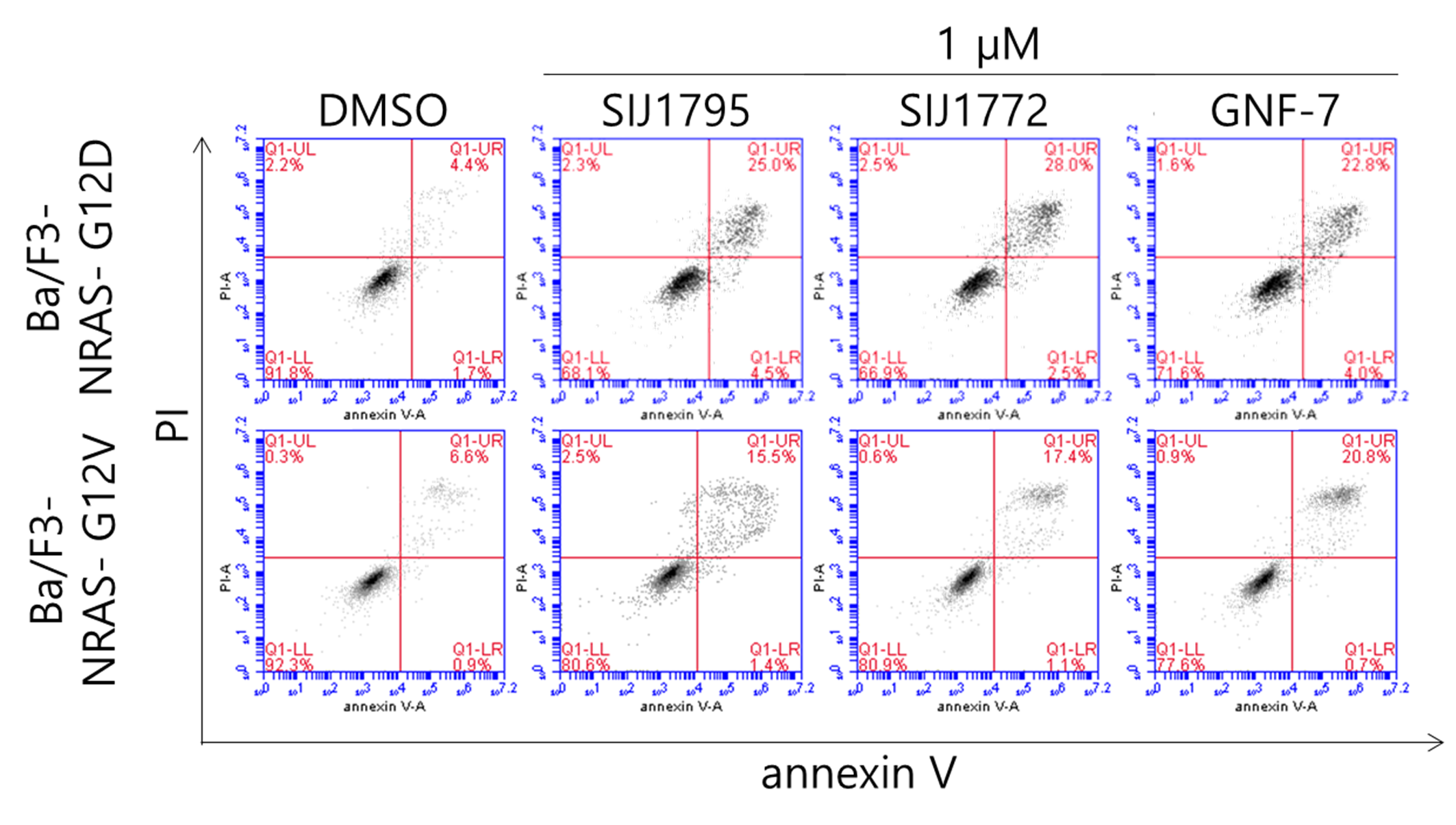

Supplement: Supplementary Figure 2 — Apoptosis induction activity of SIJ1795 and SIJ1772 against cancer cells harboring mtRAS. Cells were treated with each compounds of 1 μM for 24 h and subjected to Alexa Flour 488 conjugated annexin V and propidium iodide staining. Flow cytometry analysis were performed. [file Image_2.tif]

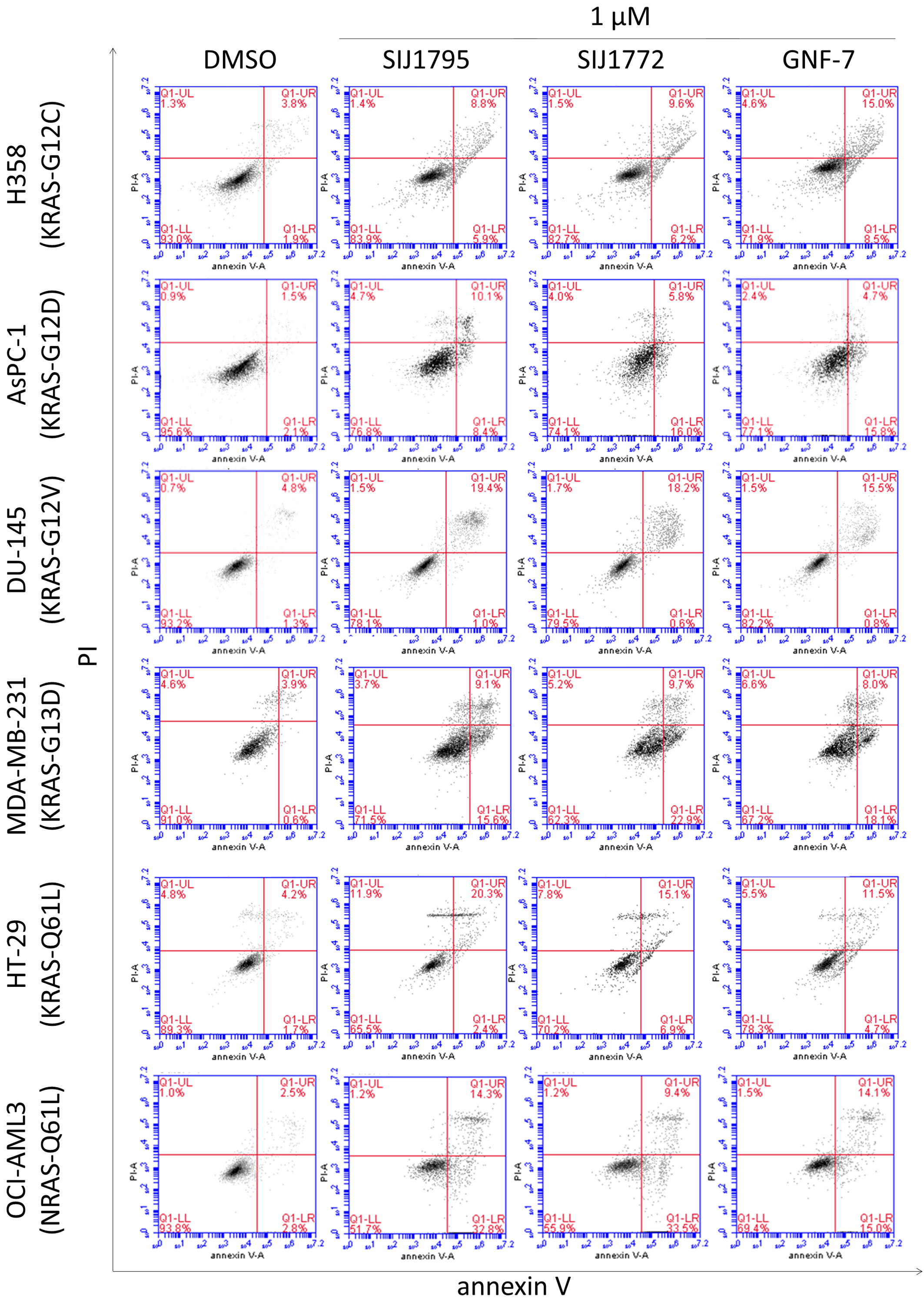

Supplement: Supplementary Figure 3 — Apoptosis induction activity of SIJ1795 and SIJ1772 cancer cells harboring mtRAS. Cells were treated with each compounds of 1 μM for 24 h and subjected to Alexa Flour 488 conjugated annexin V and propidium iodide staining. Flow cytometry analysis were performed. [file Image_3.tif]

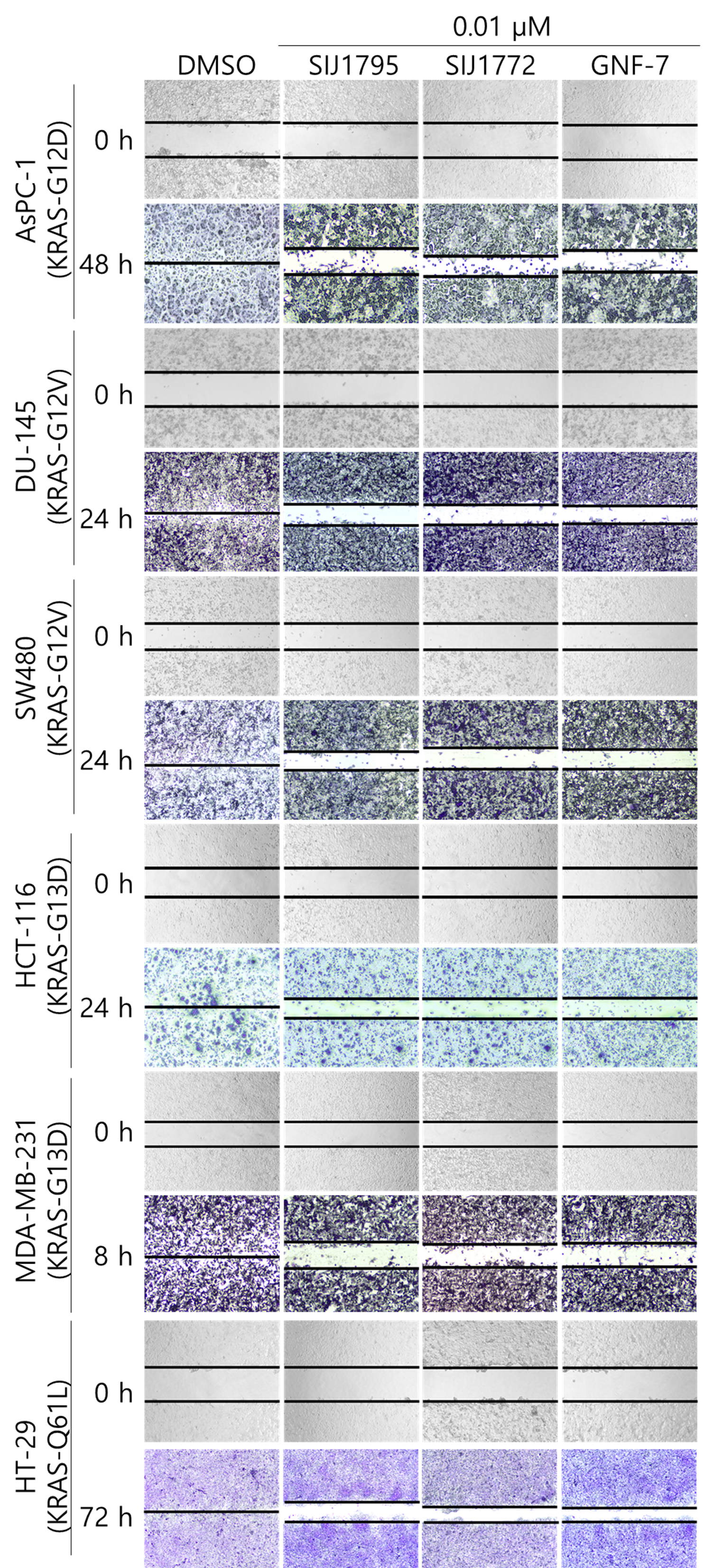

Supplement: Supplementary Figure 4 — Migration inhibitory effect of SIJ1795 and SIJ1777 cancer cells harboring mtRAS. Wound healing assay was conducted for cell migration analysis. cell monolayer was scratched and test compounds with 0.01 μM concentration were incubated for indicated time. Cells were stained with crystal violet solution and photographed with 40 × magnification. [file Image_4.tif]

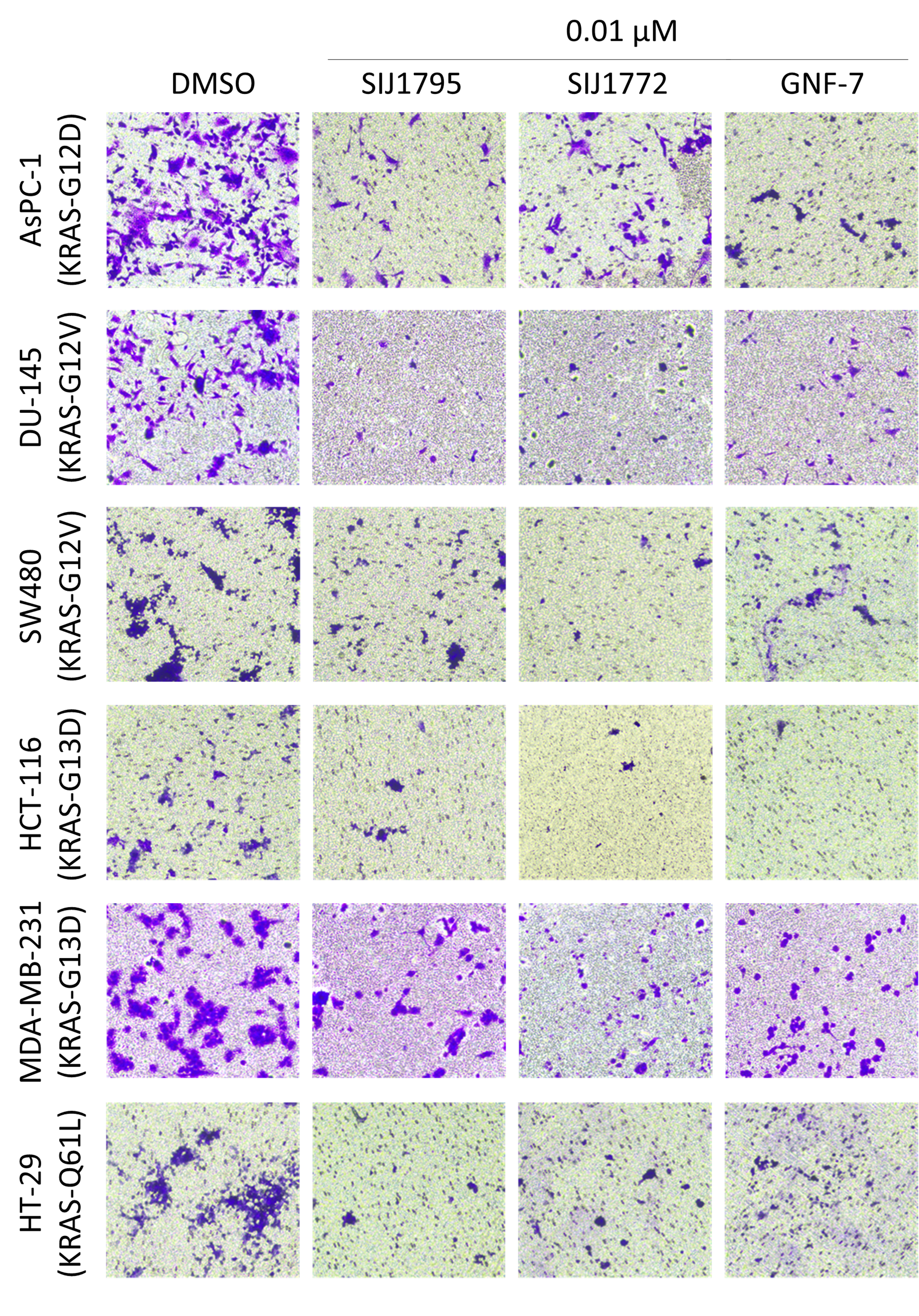

Supplement: Supplementary Figure 5 — Invasion inhibitory effect of SIJ1795 and SIJ1777 cancer cells harboring mtRAS. To analyze cell invasion ability, Boyden chamber assay was performed. Wound healing assay was conducted for cell migration analysis. Cell monolayer was scratched and test compounds with 0.01 μM concentration were incubated for indicated time. Cells were stained with crystal violet solution and photographed with 100 × magnification. [file Image_5.tif]
